# Intratumoral and peritumoral CT radiomics in predicting anaplastic lymphoma kinase mutations and survival in patients with lung adenocarcinoma: a multicenter study

**DOI:** 10.1186/s40644-025-00856-2

**Published:** 2025-03-13

**Authors:** Weiyue Chen, Guihan Lin, Ye Feng, Yongjun Chen, Yanjun Li, Jianbin Li, Weibo Mao, Yang Jing, Chunli Kong, Yumin Hu, Minjiang Chen, Shuiwei Xia, Chenying Lu, Jianfei Tu, Jiansong Ji

**Affiliations:** 1 Zhejiang Key Laboratory of Imaging and Interventional Medicine, Zhejiang Engineering Research Csaenter of Interventional Medicine Engineering and Biotechnology, Key Laboratory of Precision Medicine of Lishui City, The Fifth Affiliated Hospital of Wenzhou Medical University, Lishui, 323000 Zhejiang China; 2 School of Medicine, Clinical College of The Affiliated Central Hospital, Lishui University, Lishui, 323000 China; 3 Department of Radiology, The Sixth Affiliated Hospital of Wenzhou Medical University, Lishui, 323000 China; 4 Department of Radiology, The First Hospital of Jiaxing, Affiliated Hospital of Jiaxing University, Jiaxing, 314000 China; 5 Department of Radiology, The Affiliated People’s Hospital of Ningbo University, Ningbo, 315211 China; 6 Department of Pathology, The Fifth Affiliated Hospital of Wenzhou Medical University, Lishui, 323000 China; 7 Huiying Medical Technology Co., Ltd, Room A206, B2, Dongsheng Science and Technology Park, Haidian District, Beijing, 100192 China

**Keywords:** Lung adenocarcinoma, Anaplastic lymphoma kinase, Peritumoral, Machine learning, Prognosis

## Abstract

**Background:**

To explore the value of intratumoral and peritumoral radiomics in preoperative prediction of anaplastic lymphoma kinase (ALK) mutation status and survival in patients with lung adenocarcinoma.

**Methods:**

We retrospectively collected data from 505 eligible patients with lung adenocarcinoma from four hospitals (training and external validation sets 1–3). The CT-based radiomics features were extracted separately from the gross tumor volume (GTV) and GTV incorporating peritumoral 3-, 6-, 9-, 12-, and 15-mm regions (GPTV_3_, GPTV_6_, GPTV_9_, GPTV_12_, and GPTV_15_), and screened the most relevant features to construct radiomics models to predict ALK (+). The combined model incorporated radiomics scores (Rad-scores) of the best radiomics model and clinical predictors was constructed. Performance was evaluated using receiver operating characteristic (ROC) analysis. Progression-free survival (PFS) outcomes were examined using the Cox proportional hazards model.

**Results:**

In the four sets, 21.19% (107/505) patients were ALK (+). The GPTV_3_ radiomics model using a support vector machine algorithm achieved the best predictive performance, with the highest average AUC of 0.811 in the validation sets. Clinical TNM stage and pleural indentation were independent predictors. The combined model incorporating the GPTV_3_-Rad-score and clinical predictors achieved higher performance than the clinical model alone in predicting ALK (+) in three validation sets [AUC: 0.855 (95% CI: 0.766–0.919) vs. 0.648 (95% CI: 0.543–0.745), *P* = 0.001; 0.882 (95% CI: 0.801–0.962) vs. 0.634 (95% CI: 0.548–0.714), *P <* 0.001; 0.810 (95% CI: 0.727–0.877) vs. 0.663 (95% CI: 0.570–0.748), *P* = 0.006]. The prediction score of the combined model could stratify PFS outcomes in patients receiving ALK-TKI therapy (HR: 0.37; 95% CI: 0.15–0.89; *P* = 0.026) and immunotherapy (HR: 2.49; 95% CI: 1.22–5.08; *P* = 0.012).

**Conclusion:**

The presented combined model based on GPTV_3_ effectively mined tumor features to predict ALK mutation status and stratify PFS outcomes in patients with lung adenocarcinoma.

**Supplementary Information:**

The online version contains supplementary material available at 10.1186/s40644-025-00856-2.

## Key points

• Compared with GPTV_6_, GPTV_9_, GPTV_12_, and GPTV_15_ radiomics signatures, the GPTV_3_ radiomics signature has the highest efficiency for ALK mutation prediction.

• The combined model based on GPTV_3_ radiomics features and clinical predictors effectively predicted ALK mutation in patients with lung adenocarcinoma.

• The prediction score of the combined model was able to stratify progression-free survival in patients receiving ALK-TKI therapy and immunotherapy, which could improve the design of personalized treatment strategies.

**Supplementary Information:**

The online version contains supplementary material available at 10.1186/s40644-025-00856-2.

## Background

Lung cancer is the leading cause of cancer-related death worldwide, with non–small cell lung cancer accounting for 85% of cases, and adenocarcinoma representing the predominant histologic subtype [1]. In recent years, targeted therapy and immunotherapy have significantly improved survival rates across various stages of lung adenocarcinoma, including neoadjuvant/adjuvant therapy for resected patients and palliative treatment for advanced patients [2, 3]. Anaplastic lymphoma kinase (ALK) is an important driver gene and therapeutic target for lung adenocarcinoma. Patients with ALK mutations have better clinical outcomes to ALK-tyrosine kinase inhibitors (ALK-TKIs) compared to those with wide-type ALK, but they may not respond well to immunotherapy due to potential immunosuppression [4–6]. Accordingly, pre-treatment detection of ALK mutation status has become increasingly common, helping clinicians identify patients suited to either ALK-TKI therapy or immunotherapy and thus supporting more accurate individual treatment planning.

Currently, tissue or cytological specimens are commonly used for detecting ALK mutations; however, these methods are mostly invasive. Additionally, tumor heterogeneity can lead to unavoidable sampling errors, potentially compromising the accuracy of detection [7]. Computed tomography (CT) is the primary imaging method for the diagnosis of lung adenocarcinoma. Several conventional CT features, such as lobulated margin, solidity, and pleural indentation have been associated with ALK gene rearrangements [8–11]. However, such CT features lack objective quantitative indicators, and their evaluation is therefore highly subjective. Radiomics transforms traditional images into high-dimensional quantitative image feature data through the transformation of the original image; calculation of feature matrices can then be used to deeply explore the biological properties and heterogeneity of the image [12]. Although several studies have confirmed the feasibility of CT radiomics in predicting ALK mutation, these studies have mainly focused on the intratumoral region, ignoring the potential value of the peritumoral region in assessing mutation status [13–15]. Reports suggest that the area surrounding a tumor may contain rich biological information, including angiogenesis, lymphatic dilation, vascular invasion, and stromal reaction, characteristics that are often closely related to the biological characteristics of the tumor [16]. To date, peritumoral radiomics has been confirmed to provide additional value in differential diagnosis, lymph node metastasis prediction, prognosis, and efficacy evaluation of lung cancer [17–20]. However, there are currently few studies on the prediction of ALK mutation status using a peritumoral radiomics approach.

The purpose of our study was to evaluate the performance of CT radiomics features extracted from intratumoral and peritumoral regions in predicting ALK mutation in patients with lung adenocarcinoma. We aimed to develop and validate a stable, accurate, and non-invasive prediction model for ALK mutation status. Furthermore, we investigated the potential implications of the prediction model on progression-free survival (PFS) outcomes in patients with lung adenocarcinoma.

## Methods

### Study design and participants

**Fig. 1:**
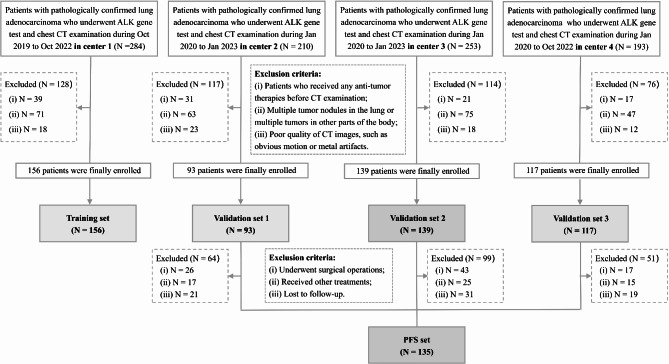
Flowchart illustrating the process of patient selection from four medical centers in this study. ALK, anaplastic lymphoma kinase; PFS, progression-free survival

This study was approved by the Institutional Review Boards, and the requirement for informed consent was waived due to retrospective nature. The study was reported according to Standards for the Reporting of Diagnostic Accuracy Studies (STARD) guidelines [21]. Data were acquired concurrently from patients at four university teaching hospitals, including the Fifth Affiliated Hospital of Wenzhou Medical University (center 1), Affiliated People’s Hospital of Ningbo University (center 2), Jiaxing No.1 Hospital (center 3), and the Sixth Affiliated Hospital of Wenzhou Medical University (center 4). After applying inclusion and exclusion criteria (Appendix E1), a total of 505 cases were included, with 156 (30.89%) in the training set, 93 (18.42%) in the validation set 1, 139 (27.52%) in the validation set 2, and 117 (23.17%) in the validation set 3. The patient recruitment process is shown in Fig. 1. Patients from the validation sets who did not meet the exclusion criteria (Appendix E2) were also included in the PFS set. ALK mutant status was confirmed by immunohistochemical staining of surgical or biopsy specimens (Appendix E3), and all patients were divided into 107 patients with ALK-positive (ALK (+)) and 398 patients with ALK-negative (ALK (-)) groups. Clinical information including age, sex, smoking history, and clinical Tumor Node Metastasis (TNM) stage [22] was obtained from medical records.

### CT acquisition and evaluation

All patients underwent non-contrast CT of the chest performed using 64-,128-, or 192-detector row scanners. The CT scanners and scanning parameters used for four hospitals are shown in Table S1. Two diagnosticians with more than 10 years of experience in chest diagnosis evaluated the CT images; consensus was reached by discussion in the event of disagreement. Conventional CT features collected included margin, density, lobulation sign, spicule sign, pleural indentation sign, air bronchogram sign, and vacuole sign. The definitions and scoring rules of these conventional CT features are described in Table S2.

### Tumor contour preparation and segmentation

**Fig. 2:**
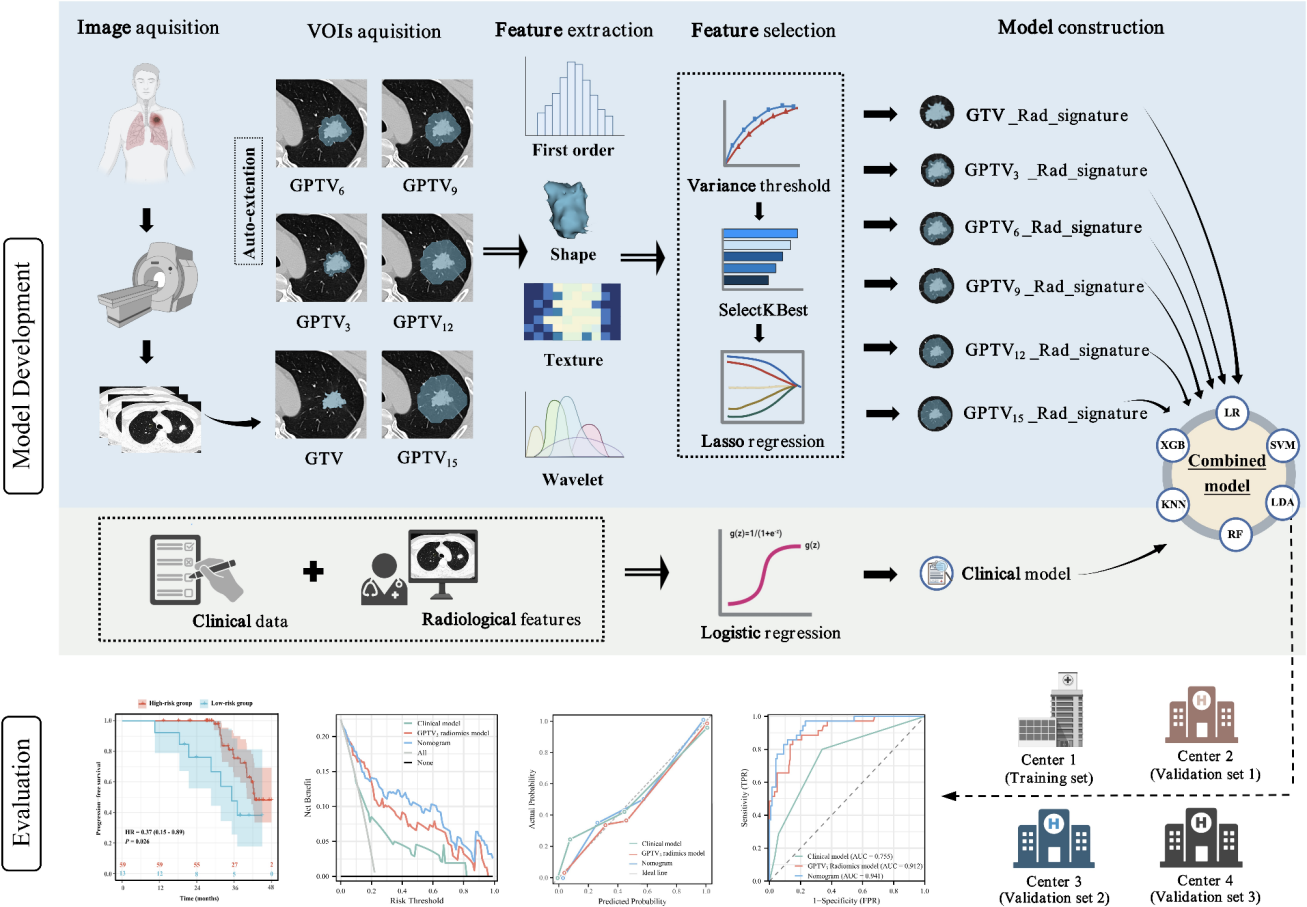
The workflow of the study. Preoperative non-contrast CT chest images of patients with lung adenocarcinoma were retrospectively collected and pre-processed, and then segmented for feature extraction. Six radiomics signatures were constructed after feature selection. The radiomics models were built using six machine learning classifiers, and the one with the best predictive performance in the validation sets was used to calculate the radiomics score (Rad-score). A clinical model was constructed using LR analysis, and a combined model incorporating the Rad-score and clinical predictors was constructed and presented as a nomogram. The performance of the nomogram was evaluated by receiver operating characteristic analysis, calibration curves, decision curve analysis, and survival curves. GTV, gross tumor volume; GPTV, gross peritumoral tumor volume; LDA, linear discriminant analysis; SVM, support vector machine; RF, random forest; KNN, k-nearest neighbor; XGBoost, eXtreme Gradient Boosting

Figure 2 shows the workflow of the study. Image analysis was performed using the platform ITK-SNAP (v3.8, http://www.itksnap.org). The gross tumor volume (GTV) was defined as the tumor identified within the visible tumor border. The region of interest (ROI) of GTV was manually outlined layer-by-layer along the tumor edge by two radiologists with more than five years of experience in thoracic diagnosis, before the 3D volume of interest (VOI) was generated. Based on previous studies [19, 23–26], we used ITK-SNAP to automatically expand the GTV outward by 3 mm, 6 mm, 9 mm, 12 mm, and 15 mm and thus obtained the ROI of the gross peritumoral tumor volume (GPTV) of GPTV_3_, GPTV_6_, GPTV_9_, GPTV_12_, and GPTV_15_ respectively. All delineated GPTV target areas included air in the lungs, pulmonary vessels, and bronchi but did not include the chest wall or mediastinum. The procedure of ROI segmentation was shown in Figure S1.

### Radiomics feature extraction and selection

After image preprocessing (Appendix E4), the open-source package “Pyradiomics” in Python was used to automatically extract quantitative radiomics features. Overall, 2804 radiomics features were extracted for each VOI, and details of these features are provided in Appendix E5. Details about intra-observer and inter-observer repeatability analysis of VOIs are described in Appendix E6.

All radiomics features were Z-score normalized ($\:f\left(x\right)=\frac{s(x-{\mu\:}_{x})}{{\delta\:}_{x}}$) to eliminate the index dimension difference. Radiomics feature selection was performed using the variance threshold method, SelectKBest method, and the Least Absolute Shrinkage and Selection Operator (LASSO) algorithm (Appendix E7). These methods were employed to identify the most relevant features for each of the six regions (GTV, GPTV_3_, GPTV_6_, GPTV_9_, GPTV_12_, and GPTV_15_) in the training set.

### Radiomics model establishment

Six independent radiomics signatures were constructed using multivariable logistic regression (LR) analysis and backward stepwise regression analysis, based on the optimal features of the six regions in the training set. The score of each case calculated from these signatures reflects the probability of ALK (+) and was named the radiomics score (Rad-score). The predictive performance of these radiomics signatures in the training and validation sets was evaluated using receiver operating characteristic (ROC) curves. The radiomics signature with the highest average area under the curve (AUC) and accuracy values in the validation sets was subsequently used to construct the optimal radiomics model [27–29]. Six machine learning algorithms were used to screen the best classifier (Appendix E8).

### Clinical and combined model construction and evaluation

Univariate LR analysis was performed to assess the association between clinical-radiological characteristics and ALK mutant status, and clinical predictors with *P* < 0.1 were included in the multivariate LR analysis to establish the clinical model. To determine the overall impact of radiomics features, a combined model and corresponding nomogram integrating independent predictors and the Rad-score of the best radiomics model were constructed using multivariate LR analysis and backward stepwise regression analysis based on the Akaike Information Criterion. The predictive performance and clinical utility of the models were assessed using ROC analyses, calibration curves, and decision curve analysis (DCA), as detailed in Appendix E9. The performance of the combined model was further evaluated through subgroup analysis (e.g., sex and smoking history) to assess its robustness across the entire patient population from the three independent validation sets.

### Prognostic value of the combined model

PFS was defined as the time from the start of treatment to the progression of radiologic symptoms or death. Disease progression was defined as a 20% increase in the targeted lesion volume or the appearance of new lesions [30]. Patients in the PFS set were split into two groups: a high-risk group with a model prediction score of ≥ 0.5 and a low-risk group with a model prediction score of < 0.5. Spearman analyses were conducted to evaluate the correlation between the combined model prediction score and PFS time in both the ALK-TKI therapy and immunotherapy groups. We then calculated the hazard ratios (HRs) of PFS using Cox proportional hazards regression, comparing patients in the low and high-risk groups.

### Statistical analysis

Statistical analysis was conducted in R v4.1.2 (www.Rproject.org), MedCalc v22.0 (www.medcalc.org), and Python v3.9.7 (www.python.org). Continuous baseline characteristics were analyzed using Student’s *t*-test or the Mann–Whitney *U* test according to the results of the Kolmogorov-Smirnov test; categorical data were analyzed using the chi-squared test or Fisher’s exact analysis. The LASSO regression, LR analysis, plotting of the nomogram and calibration curves, H-L test, and DCA were performed on the R packages “glmnet”, “rms”, “generalhoslem”, and “dca.R”, respectively. Model performance comparisons were performed using the Delong test, a solid statistical approach for distinguishing between various models. We implemented the Cox proportional hazards model and Kaplan-Meier survival curves to analyze PFS. All statistical tests were two-tailed; *P* < 0.05 was considered statistically significant.

## Results

### Patient characteristics and clinical model construction

The patients’ baseline information is outlined in Table 1 and Table S3. The mean age of the entire set was 59 years; 40.59% (205/505) of patients were male. In terms of clinical TNM staging, 21.39% (108/505) patients were classed as stage IV. In the four sets, 21.19% (107/505) patients were immunohistochemically determined to be ALK (+). Overall, all sets were balanced and comparable (Table S3). Univariate and multivariate analysis showed that clinical TNM stage and pleural indentation were independent predictors of ALK (+), which were used to establish the clinical model (Appendix E10).

**Table 1:** Clinical and radiological characteristics between lung adenocarcinoma patients with ALK positive (+) and ALK negative (-) in the training and validation sets 1–3

Characteristics	Training set (*n* = 156)			Validation set 1 (*n* = 93)			Validation set 2 (*n* = 139)			Validation set 3 (*n* = 117)		
	ALK (-) (*n* = 121)	ALK (+) (*n* = 35)	*P*-value	ALK (-) (*n* = 70)	ALK (+) (*n* = 23)	*P*-value	ALK (-) (*n* = 114)	ALK (+) (*n* = 25)	*P*-value	ALK (-) (*n* = 93)	ALK (+) (*n* = 24)	*P*-value
Age, years^*^	59.44 ± 9.20	57.97 ± 11.93	0.440	61.34 ± 10.03	59.61 ± 10.83	0.482	58.11 ± 13.96	56.12 ± 15.34	0.528	60.26 ± 11.61	56.88 ± 11.72	0.207
Sex (%)			0.445			0.358			0.872			0.838
Male	50 (41.32)	17 (48.57)		32 (45.71)	8 (34.78)		43 (37.72)	9 (36.00)		37 (37.72)	9 (36.00)	
Female	71 (58.68)	18 (51.43)		38 (54.29)	15 (65.22)		71 (62.28)	16 (64.00)		56 (62.28)	15 (64.00)	
Smoking history (%)			0.254			0.643			0.239			0.221
Yes	24 (19.83)	4 (11.43)		15 (21.43)	6 (26.09)		24 (21.05)	8 (32.00)		27 (29.03)	4 (16.67)	
No	97 (80.17)	31 (88.57)		55 (78.57)	17 (73.91)		90 (78.95)	17 (68.00)		66 (70.97)	20 (83.33)	
Clinical TNM stage (%)			0.001			0.004			0.053			0.181
I-III	101 (83.47)	20 (57.14)		63 (90.00)	14 (60.87)		93 (81.58)	16 (64.00)		74 (79.57)	16 (66.67)	
IV	20 (16.53)	15 (42.86)		7 (10.00)	9 (39.13)		21 (18.42)	9 (36.00)		19 (20.43)	8 (33.33)	
Margin (%)			0.208			0.348			0.762			0.171
Unclear	38 (31.40)	15 (42.86)		29 (41.43)	7 (30.43)		33 (28.95)	8 (32.00)		22 (23.66)	9 (37.50)	
Clear	83 (68.60)	20 (57.14)		41 (58.57)	16 (69.57)		81 (79.82)	17 (68.00)		71 (76.34)	15 (62.50)	
Density (%)			0.049			0.002			0.159			0.086
Solid	36 (29.75)	18 (51.43)		13 (18.57)	13 (56.52)		23 (17.54)	9 (44.00)		23 (24.73)	10 (41.67)	
Part-solid	43 (35.54)	7 (20.00)		32 (45.71)	5 (21.74)		57 (40.35)	8 (28.00)		46 (49.46)	6 (25.00)	
Pure GGO	42 (34.71)	10 (28.57)		25 (35.72)	5 (21.74)		34 (42.11)	8 (28.00)		24 (25.81)	8 (33.33)	
Lobulation sign (%)			0.902			0.207			0.767			0.539
Yes	47 (38.84)	14 (40.00)		29 (41.43)	13 (56.52)		51 (44.74)	12 (48.00)		53 (56.99)	12 (50.00)	
No	74 (61.16)	21 (60.00)		41 (58.57)	10 (43.48)		63 (55.26)	13 (52.00)		40 (43.01)	12 (50.00)	
Spicule sign (%)			0.590			0.752			0.925			0.349
Yes	44 (36.36)	11 (31.43)		16 (22.86)	6 (26.09)		33 (28.95)	7 (28.00)		24 (25.81)	4 (16.67)	
No	77 (63.64)	24 (68.57)		54 (77.14)	17 (73.91)		81 (71.05)	18 (72.00)		69 (74.19)	20 (83.33)	
Pleural indentation (%)			< 0.001			0.002			0.064			0.036
Yes	38 (31.40)	23 (65.71)		18 (25.71)	14 (60.87)		41 (16.67)	14 (56.00)		29 (31.18)	13 (54.17)	
No	83 (68.60)	12 (34.29)		52 (74.29)	9 (39.13)		73 (83.33)	11 (44.00)		64 (68.82)	11 (45.83)	
Air bronchogram sign (%)			0.136			0.977			0.100			0.965
Yes	32 (26.45)	5 (14.29)		13 (18.57)	5 (21.74)		23 (20.18)	1 (4.00)		19 (20.43)	5 (20.83)	
No	89 (73.55)	30 (85.71)		57 (81.43)	18 (78.26)		91 (79.82)	24 (96.00)		74 (79.57)	19 (79.17)	
Vacuole sign (%)			0.521			0.095			0.973			0.395
Yes	23 (19.01)	5 (14.29)		11 (15.71)	8 (34.78)		27 (23.68)	6 (24.00)		14 (15.05)	6 (25.00)	
No	98 (80.99)	30 (85.71)		59 (84.29)	15 (65.22)		87 (76.32)	19 (76.00)		79 (84.95)	18 (75.00)	

Note:^*^Continuous values expressed as the mean ± standard deviation

**Abbreviations:** ALK, anaplastic lymphoma kinase; GGO, ground-glass opacity; TNM, Tumor Node Metastasis

### Feature extraction and selection

**Fig. 3:**
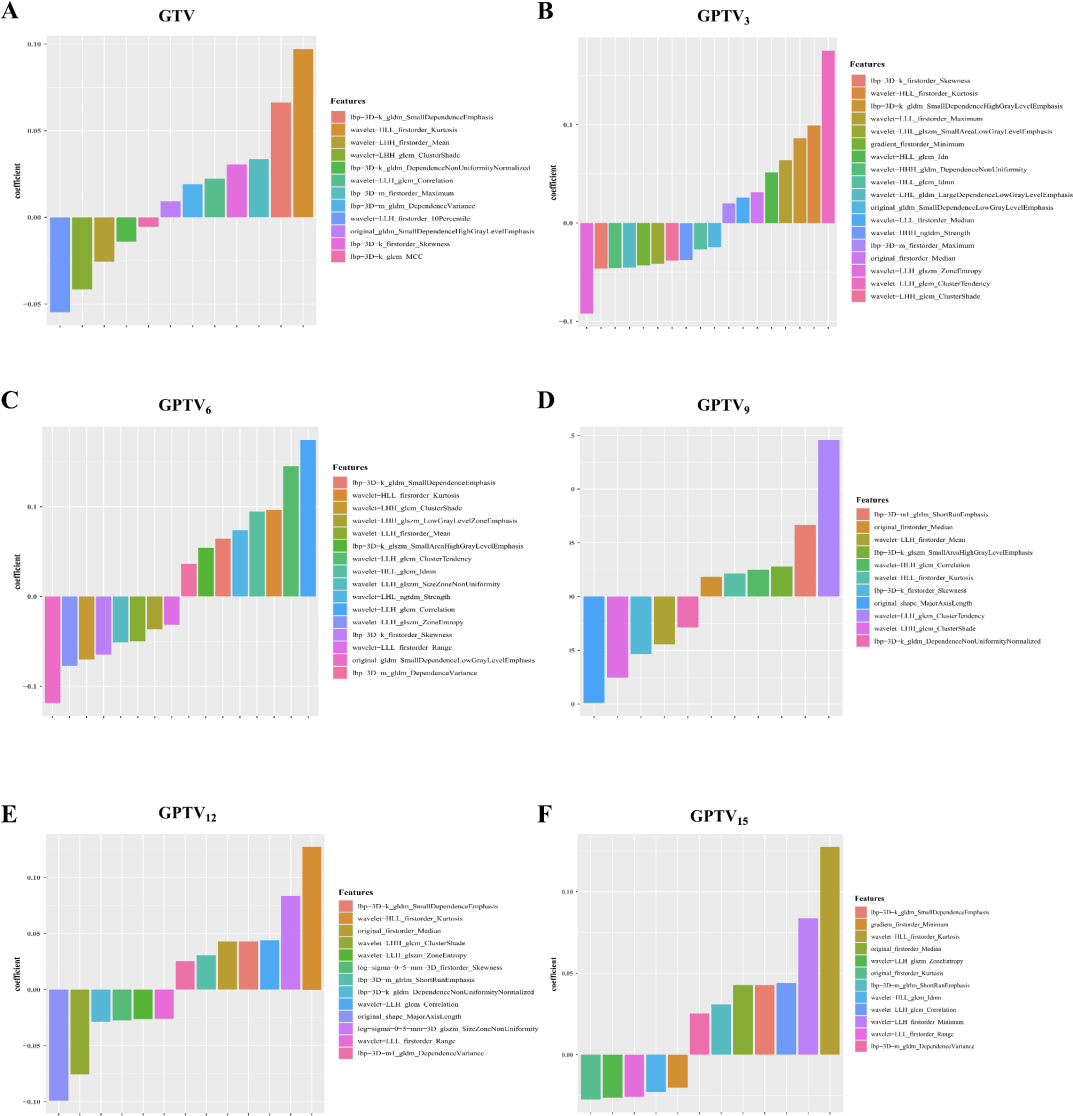
The most relevant radiomics features with nonzero coefficients were selected using the Least Absolute Shrinkage and Selection Operator (LASSO) regression analysis for each of the six regions (GTV, GPTV_3_, GPTV_6_, GPTV_9_, GPTV_12_, and GPTV_15_) in the training set. GTV, gross tumor volume; GPTV, gross peritumoral tumor volume

In total, 2804 radiomics features were extracted from each of the GTV, GPTV_3_, GPTV_6_, GPTV_9_, GPTV_12_, and GPTV_15_ VOIs; good reproducibility, with intra-observer and inter-observer ICCs > 0.80, was found for 2495 (88.98%), 2440 (87.02%), 2409 (85.91%), 2513 (89.62%), 2489 (88.77%), and 2502 (89.23%) features, respectively. For each VOI, after the process of feature selection, LASSO was finally applied to determine the optimal λ values for GTV, GPTV_3_, GPTV_6_, GPTV_9_, GPTV_12_, and GPTV_15_ as 0.187, 0.088, 0.079, 0.124, 0.068, and 0.059, respectively (Figure S2). Based on the optimal λ values, 12, 18, 16, 11, 12, and 13 features, respectively, were obtained for further constructing radiomics models (Fig. 3). The details of features selected for modeling and their coefficients are detailed in Table S5.

### Establishment of radiomics model

Following selection, we constructed feature-specific radiomics signatures using six regions and then used ROC analysis to assess the diagnostic performance of these signatures in the training and validation sets (Table 2 and Figure S3), and the results of DeLong’s tests are shown in Figure S4. Among signatures for all regions, the GPTV_3_ radiomics signature performed best, with an average AUC of 0.778 and average accuracy of 72.20% in the validation sets (Table S6). Based on the best radiomics signature model, we used six ML algorithms to improve model performance (Table 3 and Figure S5), and the results of DeLong’s tests are detailed in Figure S6. The results showed that the support vector machine (SVM) model had the best performance, with the highest average AUC of 0.811 and the highest average accuracy of 77.17% in the validation sets (Table S7). Therefore, the SVM-based GPTV_3_ radiomics model was selected as the best radiomics model and used to calculate the GPTV_3_-Rad-score.

### Development, evaluation, and clinical utility of combined model

**Fig. 4:**
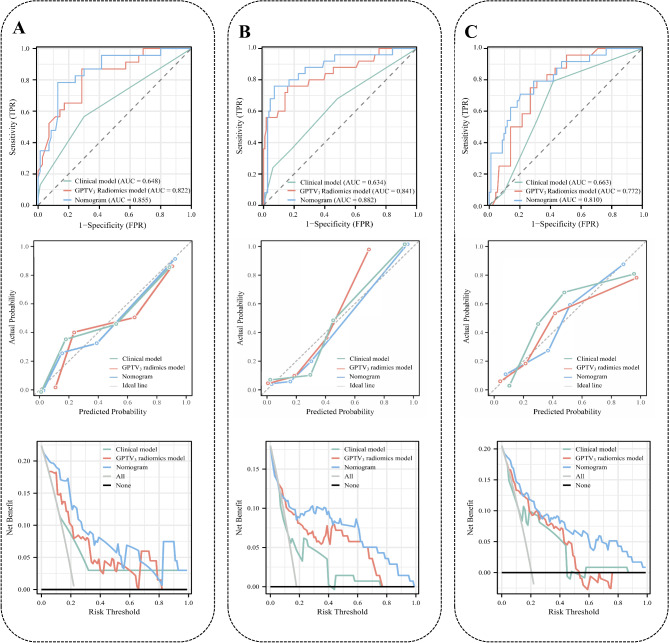
The performance of the clinical model, GPTV_3_ radiomics model and nomogram was evaluated in the validation sets. The receiver operating characteristic curves, calibration curves, and decision curve analysis curves of different models in the validation set 1 (**A**), validation set 2 (**B**), and validation set 3 (**C**). AUC, area under the curve; GPTV, gross peritumoral tumor volume

**Fig. 5:**
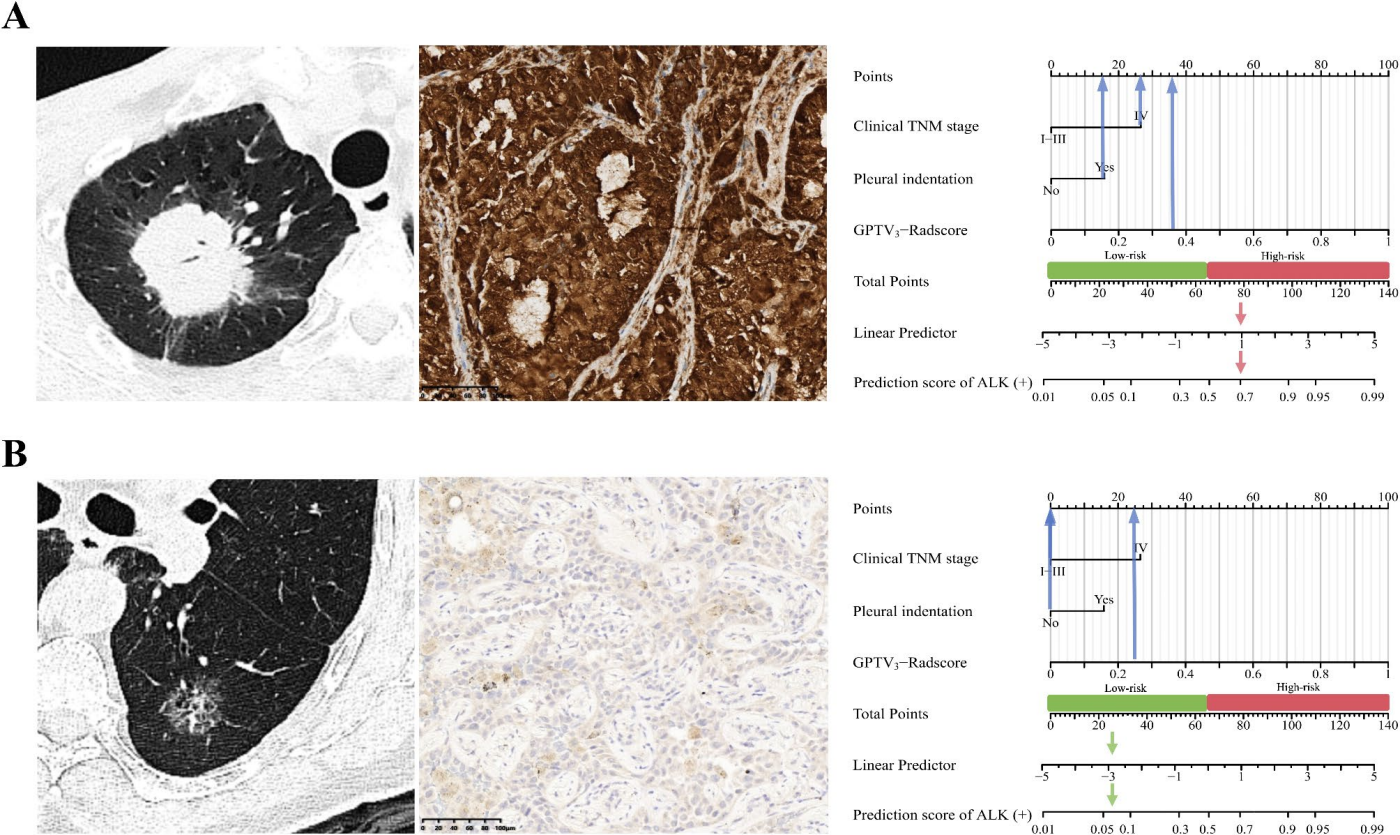
Two instances of using the nomogram to predict ALK status in lung adenocarcinoma patients. (**A**) Case 1: A 63-year-old man with a diagnosis of adenocarcinoma, clinical IV stage, manifested as a well-circumscribed solid nodule in the left upper lobe with pleural indentation sign. Immunohistochemical (IHC) staining (×200) suggests the presence of granular cytoplasmic staining, confirming ALK (+). Vertical lines of each variable were drawn in the nomogram, and to obtain the GPTV_3_-Rad-score was 0.36. After summing all variables’ points the total was 78.74, and the graph revealed that the risk of ALK (+) was approximately 70%. (**B**) Case 2: A 52-year-old man with a diagnosis of adenocarcinoma, clinical I stage, manifesting as an ill-defined mixed-density nodule in the right upper lobe. IHC staining (×200) suggests no obvious stained cells, confirming ALK (-). Vertical lines of each variable were drawn in the nomogram. The GPTV_3_-Rad-score was 0.25. After summing all variables’ points the total was 25.00, and the graph revealed that the risk of ALK (+) was approximately 7%. ALK, anaplastic lymphoma kinase; GPTV, gross peritumoral tumor volume

The GPTV_3_-Radscore, clinical TNM stage, and pleural indentation were incorporated into the combined model (Table S8) and then visualized as a nomogram (Figure S7). The radiomics nomogram was evaluated in the training set (Figure S8), and validation sets 1 (Fig. 4A), 2 (Fig. 4B), and 3 (Fig. 4C). The detailed diagnostic performance of the three models is summarized in Table 4, and the results of DeLong’s tests are listed in Table S9. Notably, the incorporation of the GPTV_3_-Radscore led to a significant increase in the AUC values compared to the clinical model in validation sets 1 to 3 from 0.648 [95% confidence interval (CI): 0.543–0.745] to 0.855 (95% CI: 0.766–0.919) (Z = 3.288, *P* = 0.001), 0.634 (95% CI: 0.548–0.714) to 0.882 (95% CI: 0.801–0.962) (Z = 3.961, *P <* 0.001), and 0.663 (95% CI: 0.570–0.748) to 0.810 (95% CI: 0.727–0.877) (Z = 2.751, *P* = 0.006), respectively. The calibration curves demonstrate that predicted ALK status had good agreement with actual observations. The Hosmer-Lemeshow test showed that the nomogram fit well (*P* > 0.05). The DCA results showed that the nomogram achieved a better net benefit than clinical and GPTV_3_ radiomics models. Table S10 lists the corresponding scores of each variable and the formula to calculate the prediction score in the nomogram. Figure 5 presents two instances that illustrate the nomogram’s clinical utility.

**Table 2:** Diagnostic performance of radiomics models for ALK mutation status in the training and validation sets 1–3

Models	Cohorts	AUC (95% CI)	Sensitivity (%) (95% CI)	Specificity (%) (95% CI)	Accuracy (%) (95% CI)	PPV (%) (95% CI)	NPV (%) (95% CI)
GTV	Training set	0.730 (0.654–0.798)	68.57 (24/35) (52.17–84.97)	67.77 (82/121) (59.44–76.10)	67.95 (106/156) (60.63–75.27)	38.10 (24/63) (26.10-50.09)	88.17 (82/93) (81.61–94.74)
	Validation set 1	0.652 (0.546–0.748)	73.91 (17/23) (52.17–84.97)	57.14 (40/70) (45.55–68.74)	61.29 (57/93) (51.39–71.19)	36.17 (17/47) (22.43–49.91)	86.96 (40/46) (77.22–96.69)
	Validation set 2	0.693 (0.609–0.768)	72.00 (18/25) (54.40–89.60)	62.28 (71/114) (53.38–71.18)	64.03 (89/139) (56.05–72.01)	29.51 (18/61) (18.06–40.95)	91.03 (71/78) (84.68–97.37)
	Validation set 3	0.669 (0.576–0.753)	75.00 (18/24) (57.68–92.32)	59.14 (55/93) (49.15–69.13)	62.39 (73/117) (53.62–71.17)	32.14 (18/56) (19.91–44.37)	90.16 (55/61) (82.69–97.64)
GPTV_3_	Training set	0.872 (0.810–0.920)	82.86 (29/35) (70.37–95.34)	80.99 (98/121) (74.00-87.98)	81.41 (127/156) (75.31–87.52)	55.77 (29/52) (42.27–69.27)	94.23 (98/104) (89.75–98.71)
	Validation set 1	0.779 (0.681–0.858)	78.26 (18/23) (61.40-95.12)	68.57 (48/70) (57.70-79.45)	70.97 (66/93) (61.74–80.19)	45.00 (18/40) (29.58–60.42)	90.57 (48/53) (82.70-98.44)
	Validation set 2	0.803 (0.727-865)	76.00 (19/25) (59.26–92.74)	75.44 (86/114) (67.54–83.34)	75.54 (105/139) (68.39–82.69)	40.43 (19/47) (26.40-54.46)	93.48 (86/92) (88.43–98.52)
	Validation set 3	0.752 (0.664–0.827)	75.00 (18/24) (57.68–92.32)	68.82 (64/93) (59.40-78.23)	70.09 (82/117) (61.79–78.38)	38.30 (18/47) (24.40–52.20)	91.43 (64/70) (84.87–97.99)
GPTV_6_	Training set	0.766 (0.691–0.830)	80.00 (28/35) (66.75–93.25)	70.25 (85/121) (62.10-78.39)	72.44 (113/156) (65.42–79.45)	43.75 (28/64) (31.60–55.90)	92.39 (85/92) (86.97–97.81)
	Validation set 1	0.671 (0.566–0.765)	69.57 (16/23) (50.76–88.37)	65.71 (46/70) (54.59–76.83)	66.67 (62/93) (57.09–76.25)	40.00 (16/40) (24.82–55.18)	86.79 (46/53) (77.68–95.91)
	Validation set 2	0.760 (0.680–0.828)	76.00 (19/25) (59.26–92.74)	68.42 (78/114) (59.89–76.95)	69.78 (97/139) (62.15–77.42)	34.55 (19/55) (21.98–47.11)	92.86 (78/84) (87.35–98.36)
	Validation set 3	0.726 (0.636–0.805)	79.17 (19/24) (62.92–95.41)	61.29 (57/93) (51.39–71.19)	64.96 (76/117) (56.31–73.60)	34.55 (19/55) (21.98–47.11)	91.94 (57/62) (85.16–98.71)
GPTV_9_	Training set	0.809 (0.739–0.868)	74.29 (26/35) (59.81–98.71)	76.03 (92/121) (68.43–83.64)	75.64 (118/156) (68.91–82.38)	47.27 (26/55) (34.08–60.47)	91.09 (92/101) (85.53–96.65)
	Validation set 1	0.713 (0.610–0.802)	82.61 (19/23) (67.12–98.10)	65.71 (46/70) (54.59–76.83)	69.89 (65/93) (60.57–79.22)	44.19 (19/43) (29.34–59.03)	92.00 (46/50) (84.48–99.52)
	Validation set 2	0.725 (0.643–0.797)	72.00 (18/25) (54.40–89.60)	67.54 (77/114) (58.95–76.14)	68.35 (95/139) (60.61–76.08)	32.73 (18/55) (20.33–45.13)	91.67 (77/84) (85.76–97.58)
	Validation set 3	0.680 (0.588–0.763)	70.83 (17/24) (52.65–89.02)	65.59 (61/93) (55.94–75.25)	66.67 (78/117) (58.12–75.21)	34.69 (17/49) (21.37–48.02)	89.71 (61/68) (82.48–96.93)
GPTV_12_	Training set	0.764 (0.690–0.828)	77.14 (27/35) (63.23–91.05)	68.60 (83/121) (60.32–76.87)	70.51 (110/156) (63.36–77.67)	41.54 (27/65) (29.56–53.52)	91.21 (83/91) (85.39–97.03)
	Validation set 1	0.645 (0.539–0.741)	73.91 (17/23) (55.97–91.86)	67.14 (47/70) (56.14–78.15)	68.82 (64/93) (59.40-78.23)	42.50 (17/40) (27.18–57.82)	88.68 (47/53) (80.15–97.21)
	Validation set 2	0.690 (0.606–0.766)	80.00 (20/25) (64.32–95.68)	57.02 (65/114) (47.93–66.11)	61.15 (85/139) (53.05–69.25)	28.99 (20/69) (18.28–39.69)	92.86 (65/70) (86.82–98.89)
	Validation set 3	0.633 (0.539–0.720)	62.50 (15/24) (43.13–81.87)	66.67 (62/93) (57.09–76.25)	65.81 (77/117) (57.22–74.41)	32.61 (15/46) (19.06–46.16)	87.32 (62/71) (79.58–95.06)
GPTV_15_	Training set	0.727 (0.650–0.795)	71.43 (25/35) (56.46–86.40)	68.60 (83/121) (60.32–76.87)	69.23 (108/156) (61.99–76.47)	39.68 (25/63) (27.60-51.76)	89.25 (83/93) (82.95–95.54)
	Validation set 1	0.629 (0.522–0.727)	60.87 (14/23) (40.92–80.82)	64.29 (45/70) (53.06–75.51)	63.44 (59/93) (53.65–73.23)	35.90 (14/39) (20.84–50.95)	83.33 (45/54) (73.39–93.27)
	Validation set 2	0.652 (0.567–0.731)	56.00 (14/25) (36.54–75.46)	67.54 (77/114) (58.95–76.14)	65.47 (91/139) (57.56–73.37)	27.45 (14/51) (15.20–39.70)	87.50 (77/88) (80.59–94.41)
	Validation set 3	0.570 (0.484–0.670)	79.17 (19/24) (62.92–95.41)	43.01 (40/93) (32.95–53.07)	50.43 (59/117) (41.37–59.49)	26.39 (19/72) (16.21–36.57)	88.89 (40/45) (79.71–98.07)

**Abbreviations**: ALK, anaplastic lymphoma kinase; AUC, area under the curve; CI, confidence interval; GTV, gross tumor volume; GPTV, gross peritumoral tumor volume; NPV, negative predictive value; PPV, positive predictive value

**Table 3:** Diagnostic performance of six different machine learning models for ALK mutation status in the training and validation sets 1–3

Models	Cohorts	AUC (95% CI)	Sensitivity (%) (95% CI)	Specificity (%) (95% CI)	Accuracy (%) (95% CI)	PPV (%) (95% CI)	NPV (%) (95% CI)
LR	Training set	0.872 (0.810–0.920)	82.86 (29/35) (70.37–95.34)	80.99 (98/121) (74.00-87.98)	81.41 (127/156) (75.31–87.52)	55.77 (29/52) (42.27–69.27)	94.23 (98/104) (89.75–98.71)
	Validation set 1	0.779 (0.681–0.858)	78.26 (18/23) (61.40-95.21)	68.57 (48/70) (57.70-79.45)	70.97 (66/93) (61.74–80.19)	45.00 (18/40) (29.58–60.42)	90.57 (48/53) (82.70-98.44)
	Validation set 2	0.803 (0.727-865)	76.00 (19/25) (59.26–92.74)	75.44 (86/114) (67.54–83.34)	75.54 (105/139) (68.39–82.69)	40.43 (19/47) (26.40-54.46)	93.48 (86/92) (88.43–98.52)
	Validation set 3	0.752 (0.664–0.827)	75.00 (18/24) (57.68–92.32)	68.82 (64/93) (59.40-78.23)	70.09 (82/117) (61.79–78.38)	38.30 (18/47) (24.40–52.20)	91.43 (64/70) (84.87–97.99)
RF	Training set	0.933 (0.881–0.967)	77.14 (27/35) (63.23–91.05)	93.39 (113/121) (88.96–97.82)	89.74 (140/156) (84.98–94.50)	77.14 (27/35) (63.23–91.05)	93.39 (113/121) (88.96–97.82)
	Validation set 1	0.673 (0.568–0.767)	30.44 (7/23) (11.63–49.24)	94.29 (66/70) (88.85–99.72)	78.50 (73/93) (70.14–86.85)	63.64 (7/11) (35.21–92.06)	80.49 (66/82) (71.91–89.07)
	Validation set 2	0.626 (0.540–0.706)	56.00 (14/25) (36.54–75.46)	64.91 (74/114) (56.15–73.67)	63.31 (88/139) (55.30-71.32)	25.93 (14/54) (14.24–37.61)	87.06 (74/85) (79.92–94.19)
	Validation set 3	0.672 (0.580–0.756)	62.50 (15/24) (43.13–81.87)	73.12 (68/93) (64.11–82.31)	70.94 (83/117) (62.71–79.17)	37.50 (15/40) (22.50–52.50)	88.31 (68/77) (81.14–95.49)
SVM	Training set	0.912 (0.855–0.951)	85.71 (30/35) (74.12–97.31)	85.12 (103/121) (78.78–91.46)	85.26 (133/156) (79.69–90.82)	62.50 (30/48) (48.80–76.20)	95.37 (103/108) (48.80–76.20)
	Validation set 1	0.822 (0.729–0.894)	86.96 (20/23) (67.87–95.46)	71.43 (50/70) (60.85–82.01)	75.27 (70/93) (66.50-84.04)	50.00 (20/40) (34.50–65.50)	94.34 (50/53) (84.63–98.06)
	Validation set 2	0.841 (0.769–0.897)	76.00 (19/25) (59.26–92.74)	84.21 (96/114) (77.52–90.90)	82.73 (115/139) (76.45–89.20)	51.35 (19/37) (35.25–67.46)	94.12 (96/102) (89.55–98.68)
	Validation set 3	0.771 (0.685–0.844)	75.00 (18/24) (57.68–92.32)	73.12 (68/93) (64.11–82.31)	73.50 (86/117) (65.51–81.50)	41.86 (18/43) (27.11–56.61)	91.89 (68/74) (85.67–98.11)
KNN	Training set	0.806 (0.735–0.864)	80.00 (28/35) (66.75–93.25)	71.07 (86/121) (62.99–79.15)	73.08 (114/156) (66.12–80.04)	44.44 (28/63) (32.17–56.71)	92.47 (86/93) (87.11–97.84)
	Validation set 1	0.752 (0.652–0.836)	86.96 (20/23) (67.87–95.46)	55.71 (39/70) (53.65–73.23)	63.44 (59/93) (55.29–71.32)	39.22 (20/51) (25.82–52.62)	92.86 (39/42) (80.99–97.54)
	Validation set 2	0.675 (0.590–0.752)	76.00 (19/25) (56.57–88.50)	61.40 (70/114) (52.47–70.34)	64.03 (89/139) (56.05–72.01)	30.16 (19/63) (18.83–41.49)	92.11 (70/76) (86.04–98.17)
	Validation set 3	0.728 (0.638–0.806)	75.00 (18/24) (57.68–92.32)	60.22 (56/93) (50.27–70.16)	63.25 (74/117) (54.51–71.98)	32.73 (18/55) (20.33–45.13)	90.32 (56/62) (82.96–97.68)
LDA	Training set	0.746 (0.670–0.812)	80.00 (28/35) (66.75–93.25)	76.86 (93/121) (69.35–84.37)	77.56 (121/156) (71.02–84.11)	50.00 (28/56) (34.50–65.50)	93.00 (93/100) (88.00–98.00)
	Validation set 1	0.640 (0.534–0.737)	52.17 (12/23) (32.96–70.76)	77.14 (54/70) (67.31–86.98)	70.97 (66/93) (61.74–80.19)	42.86 (12/28) (26.51–60.93)	83.08 (54/65) (73.96–92.19)
	Validation set 2	0.714 (0.632–0.788)	68.00 (17/25) (48.41–82.79)	71.93 (82/114) (63.68–80.18)	71.22 (99/139) (63.70-78.75)	34.69 (17/49) (21.37–48.02)	91.11 (82/90) (85.23–96.99)
	Validation set 3	0.655 (0.562–0.740)	70.83 (17/24) (50.83–85.09)	62.37 (58/93) (52.52–72.21)	64.10 (75/117) (55.41–72.80)	32.69 (17/52) (19.94–45.44)	89.23 (58/65) (81.69–96.77)
XGBoost	Training set	0.861 (0.797–0.911)	80.00 (28/35) (66.75–93.25)	75.21 (91/121) (67.51–82.90)	76.28 (119/156) (69.61–82.96)	48.28 (28/58) (35.42–61.14)	92.86 (91/98) (87.76–97.96)
	Validation set 1	0.701 (0.597–0.792)	65.22 (15/23) (44.89–81.19)	68.57 (48/70) (57.70-79.45)	67.74 (63/93) (58.24–77.24)	40.54 (15/37) (24.72–56.36)	85.71 (48/56) (76.55–94.88)
	Validation set 2	0.746 (0.665–0.816)	72.00 (18/25) (52.42–85.72)	71.93 (82/114) (63.68–80.18)	71.94 (100/139) (64.47–79.41)	36.00 (18/50) (22.70–49.30)	92.14 (82/89) (86.54–97.73)
	Validation set 3	0.729 (0.639–0.807)	75.00 (18/24) (57.68–92.32)	64.52 (60/93) (54.79–74.24)	66.67 (78/117) (58.12–75.21)	35.29 (18/51) (22.18–48.41)	90.91 (60/66) (83.97–97.84)

**Abbreviations**: ALK, anaplastic lymphoma kinase; AUC, area under the curve; CI, confidence interval; LR, logistic regression; LDA, linear discriminant analysis; RF, random forest; SVM, support vector machine; KNN, k-nearest neighbor; XGBoost, eXtreme Gradient Boosting; NPV, negative predictive value; PPV, positive predictive value

**Table 4:** Diagnostic performance of prediction models for ALK mutation status in the training and validation sets 1–3

Models	Cohorts	AUC (95% CI)	Sensitivity (%) (95% CI)	Specificity (%) (95% CI)	Accuracy (%) (95% CI)	PPV (%) (95% CI)	NPV (%) (95% CI)
Clinical	Training set	0.690 (0.611–0.761)	74.29 (26/35) (59.81–88.77)	55.37 (67/121) (46.51–64.23)	59.62 (93/156) (51.92–67.32)	32.50 (26/80) (22.24–42.76)	88.16 (67/76) (80.89–95.42)
	Validation set 1	0.648 (0.543–0.745)	56.52 (13/23) (36.81–74.37)	70.00 (49/70) (59.36–80.74)	66.67 (62/93) (57.09–76.25)	38.24 (13/34) (21.90-54.57)	83.05 (49/59) (73.48–92.62)
	Validation set 2	0.634 (0.548–0.714)	68.00 (17/25) (48.41–82.79)	51.75 (59/114) (42.58–60.93)	54.68 (76/139) (46.40-62.95)	23.61 (17/72) (13.80-33.42)	88.06 (59/67) (80.30-95.82)
	Validation set 3	0.663 (0.570–748)	79.17 (19/24) (59.93–90.76)	58.06 (54/93) (48.04–68.10)	62.39 (73/117) (52.36–71.17)	32.76 (19/58) (20.68–44.84)	91.53 (54/59) (84.42–98.63)
GPTV_3_ Radiomics	Training set	0.912 (0.855–0.951)	85.71 (30/35) (74.12–97.31)	85.12 (103/121) (78.78–91.46)	85.26 (133/156) (79.69–90.82)	62.50 (30/48) (48.80–76.20)	95.37 (103/108) (48.80–76.20)
	Validation set 1	0.822 (0.729–0.894)	86.96 (20/23) (67.87–95.46)	71.43 (50/70) (60.85–82.01)	75.27 (70/93) (66.50-84.04)	50.00 (20/40) (34.50–65.50)	94.34 (50/53) (84.63–98.06)
	Validation set 2	0.841 (0.769–0.897)	76.00 (19/25) (59.26–92.74)	84.21 (96/114) (77.52–90.90)	82.73 (115/139) (76.45–89.20)	51.35 (19/37) (35.25–67.46)	94.12 (96/102) (89.55–98.68)
	Validation set 3	0.771 (0.685–0.844)	75.00 (18/24) (57.68–92.32)	73.12 (68/93) (64.11–82.31)	73.50 (86/117) (65.51–81.50)	41.86 (18/43) (27.11–56.61)	91.89 (68/74) (85.67–98.11)
Nomogram	Training set	0.951 (0.904–0.979)	85.71 (30/35) (74.21–97.31)	92.56 (112/121) (88.05–97.56)	91.03 (142/156) (87.51–95.45)	76.92 (30/39) (62.50-88.39)	95.73 (112/117) (92.81–98.49)
	Validation set 1	0.855 (0.766–0.919)	78.26 (18/23) (61.40-95.12)	87.14 (61/70) (77.96–94.74)	84.95 (79/93) (76.13–91.52)	66.67 (18/27) (47.82–84.35)	92.42 (61/66) (86.33–97.77)
	Validation set 2	0.882 (0.801–0.962)	76.00 (19/25) (56.57–88.50)	92.98 (106/114) (87.65–97.46)	89.93 (125/139) (85.81–92.56)	70.37 (19/27) (52.82–86.82)	94.64 (106/112) (89.20-98.07)
	Validation set 3	0.810 (0.727–0.877)	70.83 (17/24) (50.83–85.09)	79.57 (74/93) (71.24–86.46)	77.78 (91/117) (69.63–85.45)	47.22 (17/36) (30.45–61.83)	91.36 (74/81) (84.99–97.13)

**Abbreviations**: ALK, anaplastic lymphoma kinase; AUC, area under the curve; CI, confidence interval; GPTV, gross peritumoral tumor volume; NPV, negative predictive value; PPV, positive predictive value

### Subgroup prediction performance of the combined model

Subgroup analyses, separately stratified by sex and smoking history, were conducted across the entire patient population from the three independent validation sets. The combined model yielded an AUC of 0.831 (95% CI: 0.741–0.921) in the male group and 0.868 (95% CI: 0.813–0.924) in the female group. The AUC was 0.877 (95% CI: 0.821–0.934) for patients with no smoking history and 0.824 (95% CI: 0.685–0.963) for patients with a smoking history. The Delong tests showed no statistically significant differences among these two subgroup analyses (sex subgroup: Z = 0.184, *P* = 0.854; smoking history subgroup: Z = 0.695, *P* = 0.489). The subgroup prediction performance and AUC curves are presented in Table S11 and Figure S9.

### Individualized prognostic stratification

**Fig. 6:**
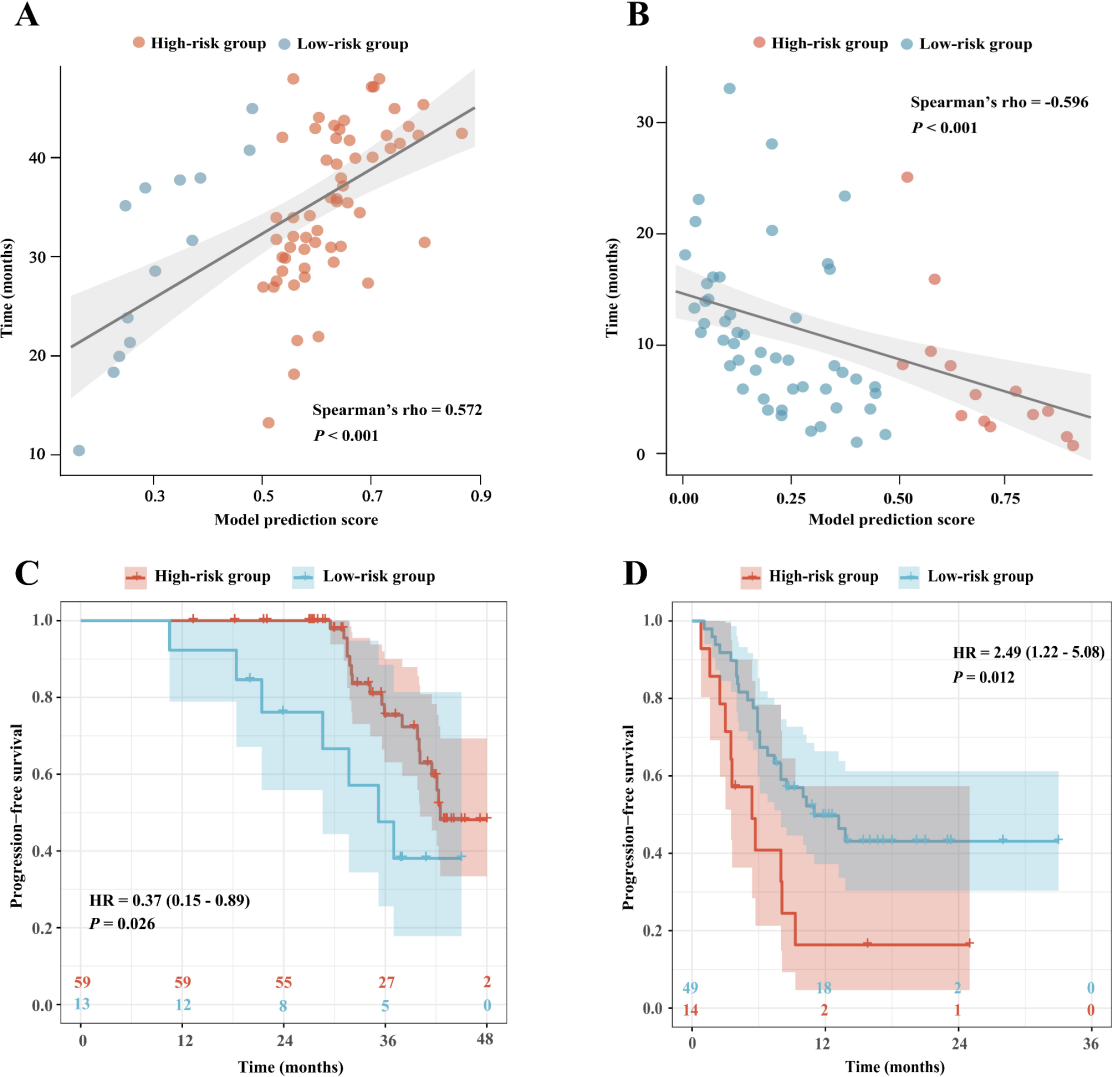
Individualized prognostic stratification of the prediction score of the combined model in ALK (+) patients undergoing ALK-TKI therapy and ALK (-) patients receiving immunotherapy. The correlation between the prediction score and the progression-free survival time in the ALK-TKI therapy group (**A**) and immunotherapy group (**B**). The progression-free survival stratification of the model prediction score in the ALK-TKI therapy group (**C**) and immunotherapy group (**D**). ALK, anaplastic lymphoma kinase; HR, hazard ratio; TKI, tyrosine kinase inhibitor

The PFS data comprised patients who received ALK-TKI therapy (*n* = 72) and patients who received immunotherapy (*n* = 63). In the ALK-TKI therapy group, the median PFS was estimated to be 42.30 (95% CI: 39.40, 45.20) months, while in the immunotherapy group it was 8.50 (95% CI: 5.89, 11.11) months. In the ALK-TKI therapy group, a statistically significant positive correlation was observed between the model prediction score and PFS time, with a Spearman’s rho of 0.572 (*P* < 0.001) (Fig. 6A); further analysis revealed that patients in the high-risk group had a significantly longer PFS duration compared to those in the low-risk group (HR: 0.37; 95% CI: 0.15–0.89; *P* = 0.026) (Fig. 6C). Conversely, the immunotherapy group showed a statistically significant negative association between the model prediction score and PFS time, with a Spearman’s rho of -0.596 (*P* < 0.001) (Fig. 6B); individuals in the low-risk group exhibited a longer PFS time compared to those in the high-risk group (HR: 2.49; 95% CI: 1.22–5.08; *P* = 0.012) (Fig. 6D).

## Discussion

The identification of ALK mutation status can facilitate targeted implementation of appropriate therapies by surgeons and oncologists to improve patient outcomes [31, 32]. However, determining ALK status through pathological analysis typically requires invasive procedures. In this study, we quantitatively evaluated the use of intratumoral and peritumoral radiomics using preoperative CT images in predicting ALK mutation in patients with lung adenocarcinoma. The results show that radiomics signatures, including peritumoral features, were stable and accurate indicators at a close area surrounding the tumor (3–9 mm). A radiomics model based on the SVM classifier outperformed models based on other ML algorithms. A combined model, incorporating GPTV_3_ radiomics features and clinical predictors, outperformed the clinical model alone and exhibited robust predictive capabilities in subgroup analyses. Furthermore, we observed associations between the prediction score of the combined model and PFS in patients receiving ALK-TKI therapy and immunotherapy.

To our knowledge, few studies have revealed the added value of peritumoral radiomics in predicting ALK mutational status in lung adenocarcinoma. Previously, Cui et al. [33] demonstrated that a prediction model integrating intratumoral and peritumoral radiomics features performed slightly better than an intratumoral-only model. However, the difference was not statistically significant (AUC = 0.68 vs. 0.67, *P* = 0.738), indicating a limited capacity for peritumoral features to enhance predictive accuracy. Notably, this previous study only extracted radiomics features within 5 mm of the peritumoral range, potentially failing to capture information available from a broader area. Some studies [24, 26] have reported the average distance from lung adenocarcinoma to micrometastases as 2.69–3.0 mm, others suggest the peripheral extension distance of lung adenocarcinoma and lung squamous carcinoma must be increased to 8 mm and 6 mm, respectively, to cover 95% of micrometastases [23]. Other reports have found that a 3–9 mm area surrounding the tumor can provide biological information related to the heterogeneity of lung adenocarcinoma [25], while CT radiomics features identified by enlarging the tumor border externally by 15 mm were shown to be predictive for gene mutation in lung adenocarcinoma [19]. Thus, to compare the predictive performance of different peritumoral areas and determine the optimal peritumoral range, this study automatically extended outwards to 3 mm, 6 mm, 9 mm, 12 mm, and 15 mm from the GTV. Unlike previous studies, we simplified the process by removing intra-tumor masks and extracted radiomic features from combined segmentation, rather than extracting GTV and peritumoral tumor volume (PTV) separately [25, 34]. While the peritumoral microenvironment plays an important role in assessing tumor aggressiveness, the intratumoral region is highly representative of tumor proliferation and heterogeneity and should not be discarded when delineating ROIs [35]. Previous studies have found that the performance of the GPTV model is higher than that of a single GTV or PTV model, indicating that the area around the tumor can provide supplementary information to the intratumor area [20]. The GPTV model constructed in this study can therefore not only provide intra- and peritumoral biological information, but also help simplify clinical application. It is worth mentioning that our study used three independent external validation sets to verify the generalization ability of the model. As a result, our model may be more effective in identifying the differences in radiomics features between lung adenocarcinoma patients of different ALK mutation status.

Our results showed that the GPTV model, including proximal regions around the tumor, has a higher predictive value than the GTV model. This confirms that the inclusion of intratumoral components and incremental peritumoral information can significantly improve predictive performance. By comparing the diagnostic performance of different GPTV models, the optimal size was determined; the GPTV_3_ model showed the highest predictive performance, suggesting that efficiency decreases with increased distance from the tumor. This may be related to the increase in PTV, with the inclusion of structures adjacent to the nodule (such as blood vessels, fascicular opacities, and bronchi) [36]. We speculate that the presence of such structures reduces the heterogeneity of the radiomic signature, producing greater similarity between ALK (+) and ALK (-) group signatures, reducing the accuracy of differential diagnosis. This would explain the better performance of the GPTV_3_ compared to other models.

Consistent with several previous studies [13, 15, 33], the most predictive radiomics features ultimately selected in our study included a large number of wavelet transform-based features. This may be attributed to the ability of wavelet transform to decompose image data into different frequency components, reflecting the spatial heterogeneity of tumors at the cellular level, as well as conveying angiogenesis and genetic information [37]. Among these wavelet features, the “ClusterTendency” feature has the highest correlation coefficient. This value is derived from the skewness of the gray-level co-occurrence matrix. The larger the value, the more uneven the image texture distribution and the more irregular the grayscale changes; this indicates highly invasive tumor lesions. Such quantitative analysis of radiomics features can serve as a non-invasive method to reflect the biological behavior of tumors and provide a basis for precise personalized treatment of patients with lung adenocarcinoma.

Multivariable LR analysis identified clinical TNM stage and pleural indentation as independent predictors of ALK (+), measures then used to establish the clinical model in this study. The signaling pathway of the ALK fusion protein promotes the survival and migration of tumor cells [13], meaning ALK (+) lung cancer may be more prone to lymph node and distant metastasis and present at a higher clinical stage. Pleural indentation is an important indicator of local progression of lung cancer. Since ALK-mutated tumors have higher invasiveness and spread capabilities, this rapid expansion often leads to direct invasion of the pleura, causing inward pleural indentation [38]. We developed a combined model including clinical TNM stage and pleural indentation with the GPTV_3_ radiomics signature to predict ALK mutation status in patients with lung adenocarcinoma. Our constructed model showed promising results with AUCs of 0.855, 0.882, and 0.810 in the validation sets 1 to 3, respectively, significantly outperforming the clinical model. The combined model also exhibited robust predictive performance in subgroup analyses for sex and smoking status. We further visualized the combined model as an easy-to-use nomogram, which can assist clinicians in calculating the prediction score for ALK mutation status. Notably, the model not only provides a convenient, non-invasive tool for clinical use, but also effectively stratifies PFS outcomes in patients receiving ALK-TKI therapy and immunotherapy. These results indicated that our constructed model helps to accurately and quickly quantify the ALK mutation status, which is critical in identifying of lung adenocarcinoma patients suitable for ALK-TKI therapy, and also provides potential possibilities for guiding immunotherapy. The use of this tool can help clinicians develop and optimize personalized treatment strategies and enhance patient management in clinical practice.

Our study still has some limitations. First, this is a retrospective study and may have varying degrees of selection bias. Future studies should perform prospective trials to confirm these conclusions and extend their relevance to broader populations. Second, only non-contrast CT was used in this study to construct the prediction model. Further exploration of using contrast CT for ALK mutation prediction will be considered, as this may provide a more comprehensive tumor assessment and enhance predictive performance. Third, the sample was limited demographically and ethnically. We will expand the validation cohort to include a more diverse and multi-ethnic population to assess the generalizability of the model. Fourth, although radiomics features extracted through manual segmentation are more accurate, this process is time and labor intensive. As automated image segmentation technology advances, future research should focus on integrating these tools into radiomics analysis to reduce time and labor while preserving or enhancing feature extraction accuracy. Finally, this study solely divided TNM into stages I-III and IV, which may overlook the differences between patients in stages I, II, and III. Therefore, we plan to conduct more detailed validation of the model’s applicability in patients with different TNM stages in future research.

## Conclusions

In summary, GPTV_3_ radiomics signatures based on an SVM classifier can provide non-invasive biomarkers for predicting ALK mutation status in patients with lung adenocarcinoma. Additionally, a combined model incorporating GPTV_3_-Rad-score and clinical predictors can further improve predictive efficiency and stratify PFS outcomes in patients receiving ALK-TKI therapy and immunotherapy. This may serve as an important tool for the formulation of personalized treatment strategies for lung adenocarcinoma patients. We suggest that future research should investigate the biological mechanisms by which the peritumoral microenvironment influences ALK mutation status, as this could reveal new therapeutic targets and improve treatment precision for lung adenocarcinoma.

## Electronic supplementary material

Below is the link to the electronic supplementary material.

Supplementary Material 1

## Data Availability

The datasets used and/or analyzed in the current study are available from the corresponding author upon reasonable request.

## Abbreviations

AUC: Area under the curve
ALK: Anaplastic lymphoma kinase
CT: Computed tomography
DCA: Decision curve analysis
GTV: Gross tumor volume
GPTV: Gross peritumoral tumor volume
HR: Hazard ratio
ICC: Intra-class correlation coefficient
KNN: K-nearest neighbors
LASSO: Least absolute shrinkage and selection operator
LDA: Linear discriminant analysis
LR: Logistic regression
PFS: Progression-free survival
Rad-score: Radiomics score
RF: Random forest
ROC: Receiver operating characteristic
SVM: Support vector machine
TNM: Tumor Node Metastasis
TKI: Tyrosine kinase inhibitor
VOI: Volume of interest
XGBoost: eXtreme Gradient Boosting
