# Supplementary material for: Intratumoral and peritumoral CT radiomics in predicting anaplastic lymphoma kinase mutations and survival in patients with lung adenocarcinoma: a multicenter study
Source: Cancer Imaging. 2025 Mar 13;25:35. doi: 10.1186/s40644-025-00856-2 (PMC11907895; doi:10.1186/s40644-025-00856-2)

**Intratumoral and peritumoral CT radiomics in predicting anaplastic lymphoma kinase mutations in patients with lung adenocarcinoma: a multicenter study**

**Supplementary Materials**

1. **Supplementary Methods**

**Appendix E1.** Inclusion and exclusion criteria for patients with lung cancer

**Appendix E2.** Exclusion criteria for the progression-free survival (PFS) set

**Appendix E3.** The procedure of pathological detection of anaplastic lymphoma kinase (ALK)

**Appendix E4.** Image preprocessing

**Appendix E5.** The details of radiomics features

**Appendix E6.** Intra-observer and inter-observer repeatability analysis

**Appendix E7.** The procedures of radiomics feature selection

**Appendix E8.** The optimal hyperparameter combinations for each machine learning classifier

**Appendix E9.** Model evaluation

**Appendix E10.** Clinical model construction

1. **Supplementary Tables**

**Table S1.** The CT scanners and scanning parameters in three centers

**Table S2.** The definitions and scoring rules for clinical characteristics and CT features

**Table S3.** Clinical and radiological characteristics of enrolled patients with lung adenocarcinoma in the training and validation sets

**Table S4.** Univariate and multivariate logistic regression analyses of clinical characteristics for predicting ALK mutation status in patient with lung adenocarcinoma in the training set

**Table S5.** The selected features for GTV, GPTV_3_, GPTV_6_, GPTV_9_, GPTV_12_ and GPTV_15_ radiomics models

**Table S6.** Diagnostic performance (AUC and ACC) of radiomics models for ALK mutation status in the training and validation sets 1-3

**Table S7.** Diagnostic performance (AUC and ACC) of six different machine learning models for ALK mutation status in the training and validation sets 1-3

**Table S8.** Multivariable logistic regression analysis for ALK mutation status in the training set

**Table S9.** The comparison of prediction models using Delong tests in the training and validation sets

**Table S10.** The corresponding score in the nomogram

**Table S11.** Subgroup prediction performance of combined model

1. **Supplementary Figures**

**Figure S1.** The procedure of ROI segmentation.

**Figure S2.** LASSO coefficient distributions of radiomics features for each VOI (**A-F**). Drawing vertical lines at the values selected using tenfold cross-validation, and features with nonzero coefficients in the LASSO regression model were the most predictive features.

**Figure S3.** The performance of GTV, GPTV_3_, GPTV_6_, GPTV_9_, GPTV_12_, and GPTV_15_ radiomics signatures in all sets. The ROC curves of six different radiomics signatures in the training set (**A**), validation set 1 (**B**), validation set 2 (**C**), and validation set 3 (**D**).

**Figure S4.** The comparison of GTV, GPTV_3_, GPTV_6_, GPTV_9_, GPTV_12_, and GPTV_15_ radiomics signatures using Delong tests (*P* value) in the training set (**A**), validation set 1 (**B**), validation set 2 (**C**), and validation set 3 (**D**).

**Figure S5.** The performance of GPTV_3_ radiomics signature with six machine learning algorithms in all sets. The ROC curves of six different machine learning models in the training set (**A**), validation set 1 (**B**), validation set 2 (**C**), and validation set 3 (**D**).

**Figure S6.** The comparison of six different machine learning models using Delong tests (*P* value) in the training set (**A**), validation set 1 (**B**), validation set 2 (**C**), and validation set 3 (**D**).

**Figure S7.** The nomogram to predict the risk of ALK (+) in patients with lung adenocarcinoma. Draw a line straight upward to the points’ axis to determine the points of each variable, add the points, and locate the final sum on the axis of total points. Finally, draw a line straight down to find the patient’s probability of ALK (+).

**Figure S8.** The performance of clinical model, GPTV_3_ radiomics model, nomogram was evaluated in the training set. The ROC curves (**A**), calibration curves (**B**), and DCA curves (**C**) of different models.

**Figure S9.** AUC of the combined model for predicting ALK (+) in different subgroups stratified by sex (A, B) and smoking status (C, D). Parentheses indicate 95% CIs for AUCs. AUC, area under the receiver-operating characteristic curve; CI, confidence interval.

**Appendix E1**

**Inclusion and exclusion criteria for patients with lung cancer**

The inclusion criteria were as follows: (i) pathologically confirmed lung adenocarcinoma; (ii) confirmed ALK mutation positive (ALK(+)) or negative (ALK(-)); (iii) undergoing chest CT examination with a slice thickness of 1mm before surgery or puncture within 2 weeks; (iv) complete clinical and pathological data.

The exclusion criteria were as follows: (i) patients who received any anti-tumor therapies before CT examination; (ii) multiple tumor nodules in the lung or multiple tumors in other parts of the body; (iii) poor quality of CT images, such as obvious motion or metal artifacts.

**Appendix E2**

**Exclusion criteria for the progression-free survival (PFS) set**

Exclusion criteria: (1) underwent surgical operations; (2) received other treatments (non-ALK-TKI treatment or non-immunotherapy); (3) lost to follow up.

**Appendix E3**

**The procedure of pathological detection of anaplastic lymphoma kinase (ALK)**

All surgical or biopsy specimens were fixed in formalin, dehydrated, paraffin-embedded and sliced into sections of uniform thickness. The sections were stained with Ventana ALK IHC kit using Roche Benchmark XT automatic immunohistochemical stainer, and the staining method was described in the instruction manual of the manufacturer’s kit. The results were independently observed and evaluated by two pathologists. Consensus was achieved through collaborative consultation whenever divergent opinions emerged. ALK positive (ALK(+)) was defined as the presence of strong granular cytoplasmic staining in tumor cells (any percentage of positive cells); and ALK negative (ALK(-)) was defined as the absence of strong granular cytoplasmic staining in tumor cells.

**Appendix E4**

**Image preprocessing**

The voxel size of VOI was resampled to 1×1×1 mm^3^ by cubic interpolation to reduce the variability of radiomics feature values arising from different voxel sizes. Hounsfeld Units (HU) were standardized by setting consistent window levels across all images, typically ranging from -1000 HU (air) to 1000 HU (bone).

**Appendix E5**

**The details of radiomics features**

A total of 2804 features were extracted for each VOI, including 14 shape and size features, 18 first-order statistical features, 75 texture features, and 2697 filter and wavelet features. The extracted radiomics features can be grouped into four groups: (1) first-order features include 18 features, also known as histogram features, which describe the distribution of voxel intensities within the volume of interest (VOI), involving energy, entropy, mean, and median; (2) shape features include 14 features, which reflect the shape and size of the region, involving mesh surface, pixel surface, perimeter, and maximum diameter; (3) texture features include 75 features, which evaluate regional heterogeneity differences, including gray level co-occurrence matrix (GLCM), gray level dependence matrix (GLDM), gray level run length matrix (GLRLM), gray level size zone matrix (GLSZM), and neighborhood gray tone difference matrix (NGTDM); (4) filter and wavelet features include 2697 features, which include the intensity and texture features derived from filter transformation and wavelet transformation of the original image, obtained by applying filters such exponential, gradient, logarithm, square, square root, lbp2D, lbp3D, and wavelet using eight frequency band combinations (low-high-low [LHL], low-high-high [LHH], high-low-low [HLL], low-low-high [LLH], high-low-high [HLH], high-high-high[HHH], high-high-low [HHL], and low-low-low [LLL]).

**Appendix E6**

**Intra-observer and inter-observer repeatability analysis**

To evaluate the intra-observer and interobserver variance, radiologist 1 repeated the identical VOIs segmentation procedure twice on 100 randomly selected patients in two weeks, and radiologist 2 independently segmented the same 100 cases. The intra-observer and inter-observer correlation coefficients (ICCs) were computed to assess the intra- and inter-observer reproducibility. The intra-observer and inter-observer ICC evaluation were conducted by the results of the two delineations of radiologist 1, and between the first delineation of radiologist 1 and radiologist 2, respectively. An ICC of greater than 0.80 was good, between 0.61-0.80 was medium, between 0.41-0.6 was average, between 0.11-0.4 was low, and below 0.1 was no consistency.

**Appendix E7**

**The procedures of radiomics feature selection**

Firstly, the variance threshold method was performed to initially select features with values of variance higher than 0.8. Secondly, the SelectKBest method was used to select features with *P* values of less than 0.05, which was based on univariate analysis with Pearson’s correlation test for continuous features and the chi-square test for categorical features. Third, the least absolute shrinkage and selection operator (LASSO) regression with 10-fold cross-validation was used to select the optimal combination of features according to the best parameter (alpha).

**Appendix E8**

**Machine learning algorithms evaluation**

Based on the radiomics features of the optimal signature, six machine learning (ML) classifier algorithms were analyzed to develop the ML-based radiomics models and select the best classifier. These included linear discriminant analysis (LDA), logistic regression (LR), support vector machine (SVM), random forest (RF), k-nearest neighbor (KNN), and eXtreme Gradient Boosting (XGBoost). Then, the diagnostic performance of the different ML-based radiomics models was evaluated using ROC curves. The models were tested on validation sets, and the ML model with the highest average AUC and accuracy values in the validation sets was selected as the best radiomics model.

The hyperparameters of the algorithm were optimized using GridSearch with ten-fold cross-validation, and the optimal hyperparameter combinations for the six classifiers are as follows:

1. LR: penalty=‘l2’, C=0.5, solver=‘lbfgs’, class_weight =‘balanced’;
2. LDA: Solver=‘svd’;
3. SVM: C=0.6, kernel=‘rbf’;
4. RF: n_estimators = 10, max_depth=3;
5. KNN: n_neighbors=5;
6. XGBoost: n_estimators=50, max_depth= 6.

**Appendix E9**

**Model evaluation**

The predictive performance of clinical and combined models were assessed using ROC analyses, with AUC values, sensitivity, specificity, and accuracy. The DeLong test was used to compare the different models’ performance. Calibration curves were drawn to assess the nomogram with the Hosmer-Lemeshow goodness-of-fit test. The clinical utility of different models was evaluated with a decision curve analysis (DCA) by measuring the net benefits under different threshold probabilities. Comparison of all models was carried out in training and validation sets.

**Appendix E10**

**Clinical model construction**

Univariable analysis results showed that clinical TNM stage, nodule density, and pleural indentation were significantly different between ALK (+) and ALK (-) groups in the training set (all *P*<0.05). Multivariable LR analysis found that clinical TNM stage (odds ratio (OR): 3.594, 95% confidence interval (CI): 1.553–8.322, *P*=0.003) and pleural indentation (OR: 2.354, 95% CI: 1.066–5.201, *P*=0.034) were independent predictors of ALK (+) in patients with lung adenocarcinoma. These characteristics were therefore used to establish the clinical model (see **Table S4** for details).

**Table S1.** The CT scanners and scanning parameters in four centers

| **Center** | **CT Scanner** | **Tube voltage**  **(**kVp) | **Tube current**  **(**mA) | **Slice thickness**  (mm) | **Scanning matrix** |
| --- | --- | --- | --- | --- | --- |
| **Center 1** | Siemens Somatom Force | 120 | Auto | 1.0 | 512×512 |
|  | Philips Brilliance iCT | 120 | Auto | 1.0 | 768×768 |
| **Center 2** | Siemens Definition AS+ | 130 | Auto | 1.0 | 512×512 |
| **Center 3** | Siemens Sensation 64 | 130 | Auto | 1.5 | 512×512 |
| **Center 4** | GE Discovery CT750 HD | 120 | Auto | 1.25 | 512×512 |

**Table S2.** The definitions and scoring rules for conventional CT features

| **CT Feature** | **Definition** | **Scoring** |
| --- | --- | --- |
| Margin | Unclear, blurred demarcation between tumor and lung parenchyma; clear, clear demarcation of the tumor from the lung parenchyma | 0, Unclear;1, Clear |
| Density | Pure ground-glass opacity (GGO), unmasking the bronchi and vascular branches below; partial solid, presence of ground-glass opacity and solid components; solid, absence of ground-glass opacity | 1, Solid; 2, Partial solid; 3, Pure ground-glass opacity (GGO) |
| Lobulation sign | Surface of the tumour that showed a wavy or scalloped configuration | 0, No; 1, Yes |
| Spicule sign | Short lines radiating from tumour margin；irregular, irregularity of tumour margin | 0, No; 1, Yes |
| Pleural indentation | Retraction of the pleura toward the tumour | 0, No; 1, Yes |
| Air bronchogram sign | Air-filled bronchi on a background of opaque airless lung | 0, No; 1, Yes |
| Vacuole sign | Gas shadow seen in tumor lesions, generally less than 5 mm | 0, No; 1, Yes |

**Table S3.** Clinical and radiological characteristics of enrolled patients with lung adenocarcinoma in the training and validation sets 1-3

| **Characteristics** | **Training set (n=156)** | **Validation set 1 (n=93)** | **Validation set 2**  **(n=139)** | **Validation set 3 (n=117)** | ***P-value*** |
| --- | --- | --- | --- | --- | --- |
| Age, years^*^ | 59.11 ± 9.86 | 60.72 ± 9.59 | 57.75 ± 14.18 | 59.56 ± 11.66 | 0.361 |
| Sex (%) |  |  |  |  | 0.742 |
| Male | 67 (42.95) | 40 (43.01) | 52 (37.41) | 46 (39.32) |  |
| Female | 89 (57.05) | 53 (56.99) | 87 (62.59) | 71 (60.68) |  |
| Smoking history (%) |  |  |  |  | 0.400 |
| Yes | 28 (17.95) | 21 (22.58) | 32 (23.02) | 31 (26.50) |  |
| No | 128 (82.05) | 72 (77.42) | 107 (76.98) | 86 (73.50) |  |
| Clinical TNM stage (%) |  |  |  |  | 0.736 |
| I-III | 121 (77.56) | 77 (82.80) | 109 (78.42) | 90 (76.92) |  |
| IV | 35 (22.44) | 16 (17.20) | 30 (21.58) | 27 (23.08) |  |
| Margin (%) |  |  |  |  | 0.237 |
| Unclear | 103 (66.03) | 57 (61.29) | 98 (70.50) | 86 (73.50) |  |
| Clear | 53 (33.97) | 36 (38.71) | 41 (29.50) | 31 (26.50) |  |
| Density (%) |  |  |  |  | 0.179 |
| Solid | 54 (34.62) | 26 (27.96) | 32 (23.02) | 33 (28.21) |  |
| Part-solid | 50 (32.05) | 37 (39.78) | 65 (46.76) | 52 (44.44) |  |
| Pure GGO | 52 (33.33) | 30 (32.26) | 42 (30.22) | 32 (27.35) |  |
| Lobulation sign (%) |  |  |  |  | 0.062 |
| Yes | 61 (39.10) | 42 (45.16) | 63 (45.32) | 65 (55.56) |  |
| No | 95 (60.90) | 51 (54.84) | 76 (54.68) | 52 (44.44) |  |
| Spicule sign (%) |  |  |  |  | 0.125 |
| Yes | 55 (35.26) | 22 (23.66) | 40 (28.78) | 28 (23.93) |  |
| No | 101 (64.74) | 71 (76.34) | 99 (71.22) | 89 (76.07) |  |
| Pleural indentation (%) |  |  |  |  | 0.784 |
| Yes | 62 (39.74) | 32 (34.41) | 55 (39.57) | 42 (35.90) |  |
| No | 94 (60.26) | 61 (65.59) | 84 (60.43) | 75 (64.10) |  |
| Air bronchogram sign (%) |  |  |  |  | 0.580 |
| Yes | 37 (23.72) | 18 (19.35) | 24 (17.27) | 24 (20.51) |  |
| No | 119 (76.28) | 75 (80.65) | 115 (82.73) | 93 (79.49) |  |
| Vacuole sign (%) |  |  |  |  | 0.520 |
| Yes | 28 (17.95) | 19 (20.43) | 33 (23.74) | 20 (17.09) |  |
| No | 128 (82.05) | 74 (79.57) | 106 (76.26) | 97 (82.91) |  |

**Note:** *Continuous values expressed as the mean±standard deviation.

**Abbreviations:** ALK, anaplastic lymphoma kinase; GGO, ground-glass opacity; TNM, Tumor Node Metastasis.

**Table S4.** Univariate and multivariate logistic regression analyses of clinical characteristics for predicting ALK mutation status in patient with lung adenocarcinoma in the training set

| **Variables** | **Comparisons** | **Univariate logistic regression** | | | **Multivariate logistic regression*** | | |
| --- | --- | --- | --- | --- | --- | --- | --- |
|  |  | **OR** | **95% CI** | ***P* value** | **OR** | **95% CI** | ***P* value** |
| Age | Continuous variable | 0.985 | 0.948 - 1.024 | 0.438 |  |  |  |
| Sex | Male *vs.* Female | 1.341 | 0.630 - 2.854 | 0.446 |  |  |  |
| Smoking history | Yes *vs.* No | 0.522 | 0.168 - 1.619 | 0.260 |  |  |  |
| Clinical TNM stage | IV *vs.* I-III | 3.787 | 1.662 - 8.630 | 0.002 | 3.594 | 1.553-8.322 | 0.003 |
| Margin | Unclear *vs.* Clear | 1.638 | 0.757 - 3.544 | 0.210 |  |  |  |
| Density | Part-solid *vs.* Solid | 0.326 | 0.122 - 0.867 | 0.025 | 0.440 | 0.158-1.227 | 0.117 |
|  | Pure GGO *vs.* Solid | 0.476 | 0.195 - 1.162 | 0.103 | 0.695 | 0.268-1.802 | 0.454 |
| Lobulation sign | Yes *vs.* No | 0.902 | 0.487 - 2.264 | 0.902 |  |  |  |
| Spicule sign | Yes *vs.* No | 0.802 | 0.359 - 1.792 | 0.591 |  |  |  |
| Pleural indentation | Yes *vs.* No | 4.186 | 1.888 - 9.285 | < 0.001 | 2.354 | 1.066-5.201 | 0.034 |
| Air bronchogram sign | Yes *vs.* No | 0.464 | 0.166 - 1.298 | 0.143 |  |  |  |
| Vacuole sign | Yes *vs.* No | 0.710 | 0.248 - 2.029 | 0.523 |  |  |  |

**Note: ***Variables found significant at *P* < 0.1 in univariate analyses were entered into multivariate analyses.

**Abbreviations:** ALK, anaplastic lymphoma kinase; OR, odds ratio; CI, confidence interval; TNM, Tumor Node Metastasis; GGO, ground-glass opacity.

**Table S5.** The selected features for GTV, GPTV_3_, GPTV_6_, GPTV_9_, GPTV_12_ and GPTV_15_ radiomics models

| Segmentation | Image type | Feature class | Feature name | Coefficient |
| --- | --- | --- | --- | --- |
| GTV | original | gldm | SmallDependenceHighGrayLevelEmphasis | 0.00921452 |
|  | wavelet-HLL | firstorder | Kurtosis | 0.097112913 |
|  | wavelet-LHH | firstorder | Mean | -0.025576829 |
|  | wavelet-LLH | firstorder | 10Percentile | -0.054642568 |
|  | wavelet-LHH | glcm | ClusterShade | -0.041650092 |
|  | wavelet-LLH | glcm | Correlation | 0.02234693 |
|  | lbp-3D-m | firstorder | Maximum | 0.033484514 |
|  | lbp-3D-k | firstorder | Skewness | 0.030532335 |
|  | lbp-3D-k | glcm | MCC | -0.00535414 |
|  | lbp-3D-m | gldm | DependenceVariance | 0.019151572 |
|  | lbp-3D-k | gldm | SmallDependenceEmphasis | 0.06637072 |
|  | lbp-3D-k | gldm | DependenceNonUniformityNormalized | -0.013975241 |
| GPTV_3_ | original | firstorder | Median | 0.031164714 |
|  | original | gldm | SmallDependenceLowGrayLevelEmphasis | -0.024653348 |
|  | gradient | firstorder | Minimum | -0.043034658 |
|  | wavelet-HLL | firstorder | Kurtosis | 0.099182956 |
|  | wavelet-HLL | glcm | Idn | 0.051341841 |
|  | wavelet-HLL | glcm | Idmn | -0.026878217 |
|  | wavelet-LLL | firstorder | Maximum | 0.063818204 |
|  | wavelet-LLL | firstorder | Median | 0.025802849 |
|  | wavelet-LHL | glszm | SmallAreaLowGrayLevelEmphasis | -0.041467775 |
|  | wavelet-LHL | gldm | LargeDependenceLowGrayLevelEmphasis | -0.04551212 |
|  | wavelet-HHH | gldm | DependenceNonUniformity | -0.045635117 |
|  | wavelet-HHH | ngtdm | Strength | -0.037699498 |
|  | wavelet-LLH | glszm | ZoneEntropy | -0.0922029 |
|  | wavelet-LLH | glcm | ClusterTendency | 0.17530255 |
|  | wavelet-LHH | glcm | ClusterShade | -0.038094347 |
|  | lbp-3D-k | firstorder | Skewness | -0.046296928 |
|  | lbp-3D-k | gldm | SmallDependenceHighGrayLevelEmphasis | 0.086117878 |
|  | lbp-3D-m | firstorder | Maximum | 0.020082462 |
| GPTV_6_ | original | gldm | SmallDependenceLowGrayLevelEmphasis | -0.118556485 |
|  | wavelet-HLL | firstorder | Kurtosis | 0.096437354 |
|  | wavelet-HLL | glcm | Idmn | 0.094762516 |
|  | wavelet-LHH | glcm | ClusterShade | -0.070088862 |
|  | wavelet-LHH | glszm | LowGrayLevelZoneEmphasis | -0.036415362 |
|  | wavelet-LLH | firstorder | Mean | -0.049837857 |
|  | wavelet-LLH | glcm | ClusterTendency | 0.144907242 |
|  | wavelet-LLH | glszm | SizeZoneNonUniformity | -0.051118552 |
|  | wavelet-LLH | glcm | Correlation | 0.1743387 |
|  | wavelet-LLH | glszm | ZoneEntropy | -0.077379618 |
|  | wavelet-LHL | ngtdm | Strength | 0.073594567 |
|  | wavelet-LLL | firstorder | Range | -0.031374895 |
|  | lbp-3D-k | firstorder | Skewness | -0.064819624 |
|  | lbp-3D-k | glszm | SmallAreaHighGrayLevelEmphasis | 0.053996121 |
|  | lbp-3D-k | gldm | SmallDependenceEmphasis | 0.064461674 |
|  | lbp-3D-m | gldm | DependenceVariance | 0.03608598 |
| GPTV_9_ | original | shape | MajorAxisLength | -0.099013279 |
|  | original | firstorder | Median | 0.018245816 |
|  | wavelet-LLH | glcm | ClusterTendency | 0.145721414 |
|  | wavelet-LLH | firstorder | Mean | -0.004436003 |
|  | wavelet-LHH | glcm | ClusterShade | -0.075480433 |
|  | wavelet-HLH | glcm | Correlation | 0.024923453 |
|  | wavelet-HLL | firstorder | Kurtosis | 0.021480165 |
|  | lbp-3D-m | glrlm | ShortRunEmphasis | 0.006655593 |
|  | lbp-3D-k | firstorder | Skewness | -0.000533924 |
|  | lbp-3D-k | glszm | SmallAreaHighGrayLevelEmphasis | 0.002786192 |
|  | lbp-3D-k | gldm | DependenceNonUniformityNormalized | -0.028622376 |
| GPTV_12_ | original | firstorder | Median | 0.042823753 |
|  | original | firstorder | Kurtosis | -0.027475458 |
|  | gradient | firstorder | Minimum | -0.020081794 |
|  | wavelet-HLL | firstorder | Kurtosis | 0.127553756 |
|  | wavelet-HLL | glcm | Idmn | -0.022934372 |
|  | wavelet-LLH | glszm | ZoneEntropy | -0.026258525 |
|  | wavelet-LLH | glcm | Correlation | 0.043936469 |
|  | wavelet-LLH | firstorder | Minimum | 0.083707957 |
|  | wavelet-LLL | firstorder | Range | -0.025878454 |
|  | lbp-3D-k | gldm | SmallDependenceEmphasis | 0.042826813 |
|  | lbp-3D-m | glrlm | ShortRunEmphasis | 0.030770287 |
|  | lbp-3D-m | gldm | DependenceVariance | 0.02541474 |
| GPTV_15_ | original | firstorder | Median | 0.042823753 |
|  | original | shape | MajorAxisLength | -0.099013279 |
|  | wavelet-HLL | firstorder | Kurtosis | 0.127553756 |
|  | wavelet-LHH | glcm | ClusterShade | -0.075480433 |
|  | wavelet-LLH | glszm | ZoneEntropy | -0.026258525 |
|  | wavelet-LLH | glcm | Correlation | 0.043936469 |
|  | wavelet-LLL | firstorder | Range | -0.025878454 |
|  | lbp-3D-k | gldm | SmallDependenceEmphasis | 0.042826813 |
|  | lbp-3D-k | gldm | DependenceNonUniformityNormalized | -0.028622376 |
|  | lbp-3D-m | glrlm | ShortRunEmphasis | 0.030770287 |
|  | lbp-3D-m | gldm | DependenceVariance | 0.02541474 |
|  | log-sigma-0-5-mm-3D | firstorder | Skewness | -0.027475458 |
|  | log-sigma-0-5-mm-3D | glszm | SizeZoneNonUniformity | 0.083707957 |

**Table S6.** Diagnostic performance (AUC and ACC) of radiomics models for ALK mutation status in the training and validation sets 1-3

| **Models** | **Training set** | | **Validation set 1** | | **Validation set 2** | | **Validation set 3** | | **Validation sets (Average)** | |
| --- | --- | --- | --- | --- | --- | --- | --- | --- | --- | --- |
|  | **AUC** | **ACC (%)** | **AUC** | **ACC (%)** | **AUC** | **ACC (%)** | **AUC** | **ACC (%)** | **AUC** | **ACC (%)** |
| GTV | 0.730 | 67.95 | 0.652 | 61.29 | 0.693 | 64.03 | 0.669 | 62.39 | 0.671 | 62.57 |
| GPTV_3_ | 0.872 | 81.41 | 0.779 | 70.97 | 0.803 | 75.54 | 0.752 | 70.09 | 0.778 | 72.20 |
| GPTV_6_ | 0.766 | 72.44 | 0.671 | 66.67 | 0.760 | 69.78 | 0.726 | 64.96 | 0.719 | 67.14 |
| GPTV_9_ | 0.809 | 75.64 | 0.713 | 69.89 | 0.725 | 68.35 | 0.680 | 66.67 | 0.706 | 68.30 |
| GPTV_12_ | 0.764 | 70.51 | 0.645 | 68.82 | 0.690 | 61.15 | 0.633 | 65.81 | 0.656 | 65.26 |
| GPTV_15_ | 0.727 | 69.23 | 0.629 | 63.44 | 0.652 | 65.47 | 0.570 | 50.43 | 0.617 | 59.78 |

**Abbreviations:** ALK, anaplastic lymphoma kinase; AUC, area under the curve; ACC, accuracy; GTV, gross tumor volume; GPTV, gross peritumoral tumor volume.

**Table S7.** Diagnostic performance (AUC and ACC) of six different machine learning models for ALK mutation status in the training and validation sets 1-3

| **Models** | **Training set** | | **Validation set 1** | | **Validation set 2** | | **Validation set 3** | | **Validation sets (Average)** | |
| --- | --- | --- | --- | --- | --- | --- | --- | --- | --- | --- |
|  | **AUC** | **ACC (%)** | **AUC** | **ACC (%)** | **AUC** | **ACC (%)** | **AUC** | **ACC (%)** | **AUC** | **ACC (%)** |
| LR | 0.872 | 81.41 | 0.779 | 70.97 | 0.803 | 75.54 | 0.752 | 70.09 | 0.778 | 72.20 |
| RF | 0.933 | 89.74 | 0.673 | 78.50 | 0.626 | 63.31 | 0.672 | 70.94 | 0.657 | 70.92 |
| SVM | 0.912 | 85.26 | 0.822 | 75.27 | 0.841 | 82.73 | 0.771 | 73.50 | 0.811 | 77.17 |
| KNN | 0.806 | 73.08 | 0.752 | 63.44 | 0.675 | 64.03 | 0.728 | 63.25 | 0.718 | 63.57 |
| LDA | 0.746 | 77.56 | 0.640 | 70.97 | 0.714 | 71.22 | 0.655 | 64.10 | 0.670 | 68.76 |
| XGBoost | 0.861 | 76.28 | 0.701 | 67.74 | 0.746 | 71.94 | 0.729 | 66.67 | 0.725 | 68.33 |

**Abbreviations:** ALK, anaplastic lymphoma kinase; AUC, area under the curve; ACC, accuracy; LR, logistic regression; LDA, linear discriminant analysis; RF, random forest; SVM, support vector machine; KNN, k-nearest neighbor; XGBoost, eXtreme Gradient Boosting.

**Table S8.** Multivariable logistic regression analysis for ALK mutation status in the training set

| **Variables** | **Comparisons** | **OR** | **95% CI** | ***P* value** |
| --- | --- | --- | --- | --- |
| Clinical TNM stage | IV *vs.* I-III | 10.551 | 2.421 - 45.974 | 0.002 |
| Pleural indentation | Yes *vs.* No | 4.662 | 1.363 - 15.945 | 0.014 |
| GPTV_3_-Rad-score | Continuous variable | 1.803 | 1.394 - 2.333 | < 0.001 |

**Abbreviations:** ALK, anaplastic lymphoma kinase; OR, odds ratio; CI, confidence interval; TNM, Tumor Node Metastasis

**Table S9.** The comparison of prediction models using Delong tests in the training and validation sets

| **Comparisons** | **Cohorts** | **Z value** | ***P* value** |
| --- | --- | --- | --- |
| Clinical model *vs.* Nomogram | Training set | 5.500 | < 0.001 |
|  | Validation set 1 | 3.288 | 0.001 |
|  | Validation set 2 | 3.961 | < 0.001 |
|  | Validation set 3 | 2.751 | 0.006 |
| GPTV_3_ radiomics model *vs.* Nomogram | Training set | 1.833 | 0.067 |
|  | Validation set 1 | 0.889 | 0.374 |
|  | Validation set 2 | 0.928 | 0.353 |
|  | Validation set 3 | 0.640 | 0.523 |
| Clinical model *vs.* GPTV_3_ radiomics model | Training set | 3.68 | < 0.001 |
|  | Validation set 1 | 2.111 | 0.035 |
|  | Validation set 2 | 2.608 | 0.009 |
|  | Validation set 3 | 1.373 | 0.170 |

**Abbreviations:** GPTV, gross peritumoral tumor volume.

**Table S10.** The corresponding score in the nomogram

| **Variables** | **Assignment** | **Categories** | **Points** |
| --- | --- | --- | --- |
| Clinical TNM stage | 0 | I-III | 0 |
|  | 1 | IV | 26.58376 |
| Pleural indentation | 0 | No | 0 |
|  | 1 | Yes | 15.7953 |
| GPTV_3_-Rad-score* | + 100 × GPTV_3_-Rad-score | | |
| Points = Clinical TNM stage + Pleural indentation + GPTV_3_-Rad-score | | | |
| **Prediction score of ALK (+)** = -1.708e-06 * points ^3 + 0.000334744 * points ^2 + -0.007373138 * points + 0.028955408 | | | |

* Continuous variable.

**Abbreviations:** PTV, peritumor volume; TNM, Tumor Node Metastasis; Rad-score, radiomics score; ALK, anaplastic lymphoma kinase.

**Table S11.** Subgroup prediction performance of combined model

| **Subgroup** | **AUC (95% CI)** | **Sensitivity (%) (95% CI)** | **Specificity (%) (95% CI)** | **Accuracy (%) (95% CI)** | **PPV (%) (95% CI)** | **NPV (%)**  **(95% CI)** |
| --- | --- | --- | --- | --- | --- | --- |
| Sex |  |  |  |  |  |  |
| Male | 0.831 (0.741-0.921) | 69.23 (18/26)  (51.49-86.97) | 89.29 (100/112)  (83.56-95.01) | 85.51 (118/138)  (79.63-91.38) | 60.00 (18/30)  (42.47-77.53) | 92.59 (100/108)  (87.65-97.53) |
| Female | 0.868 (0.813-0.924) | 84.78 (39/46)  (74.40-95.16) | 78.79 (130/165)  (72.55-85.03) | 80.09 (169/211)  (74.71-85.48) | 52.70 (39/74)  (41.33-64.08) | 94.89 (130/137)  (91.20-98.58) |
| Smoking history |  |  |  |  |  |  |
| No | 0.877 (0.821-0.934) | 87.04 (47/54)  (78.08-96.00) | 79.15 (167/211)  (73.67-84.63) | 80.75 (214/265)  (76.01-85.50) | 51.65 (47/91)  (41.38-61.92) | 95.98 (167/174)  (93.06-98.90) |
| Yes | 0.824 (0.685-0.963) | 72.22 (13/18)  (51.53-92.91) | 89.39 (59/66)  (81.97-96.82) | 85.71 (72/84)  (78.23-93.20) | 65.00 (13/20)  (44.10-85.90) | 92.19 (59/64)  (85.61-98.76) |

**Abbreviations:** AUC, area under the curve; CI, confidence interval; NPV, negative predictive value; PPV, positive predictive value.

**Figure S1.** The procedure of ROI segmentation.

**
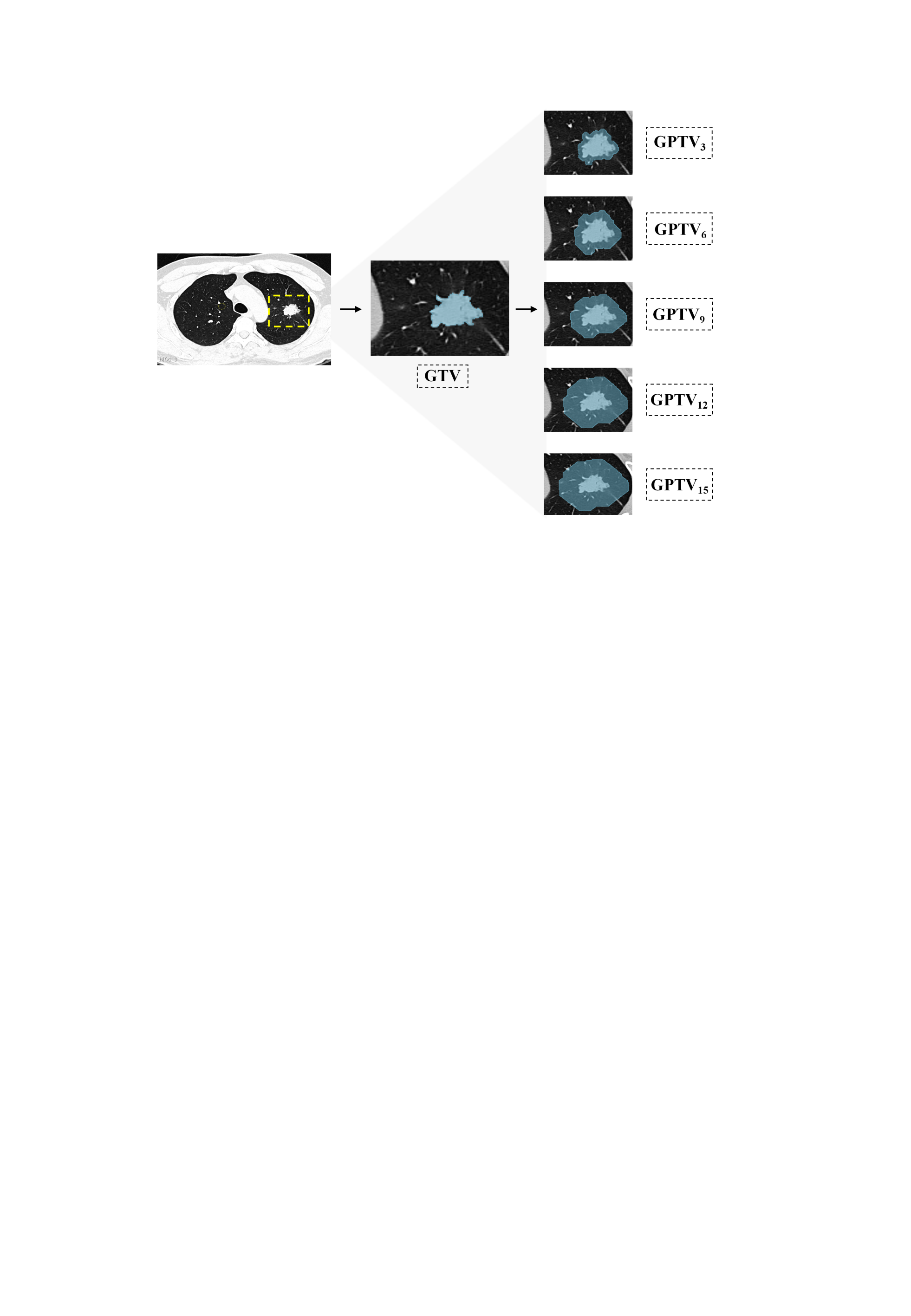
**

**Figure S2.** LASSO coefficient distributions of radiomics features for each VOI (**A-F**). Drawing vertical lines at the values selected using tenfold cross-validation, and features with nonzero coefficients in the LASSO regression model were the most predictive features.

**
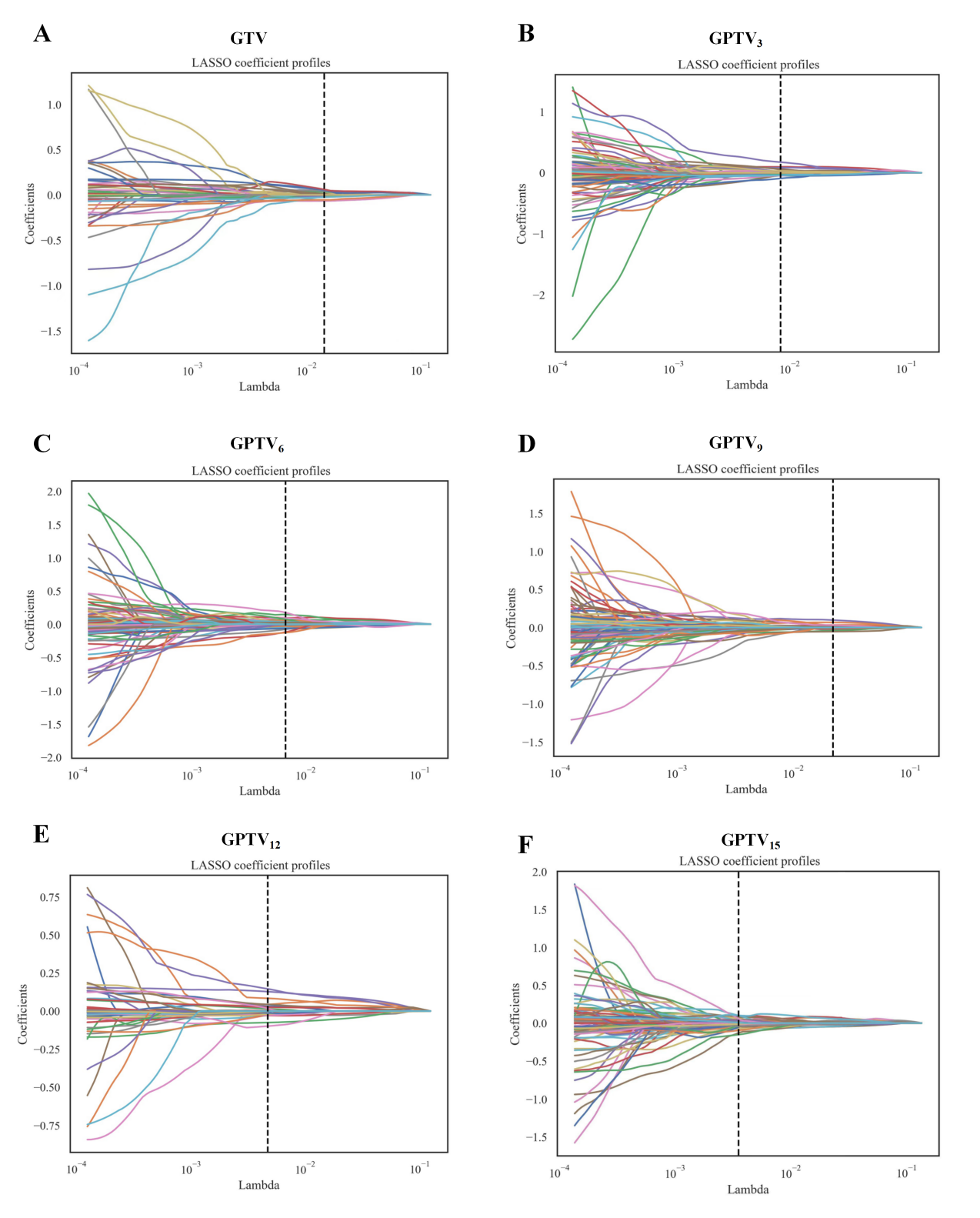
**

**Figure S3.** The performance of GTV, GPTV_3_, GPTV_6_, GPTV_9_, GPTV_12_, and GPTV_15_ radiomics signatures in all sets. The ROC curves of six different radiomics signatures in the training set (**A**), validation set 1 (**B**), validation set 2 (**C**), and validation set 3 (**D**).

**
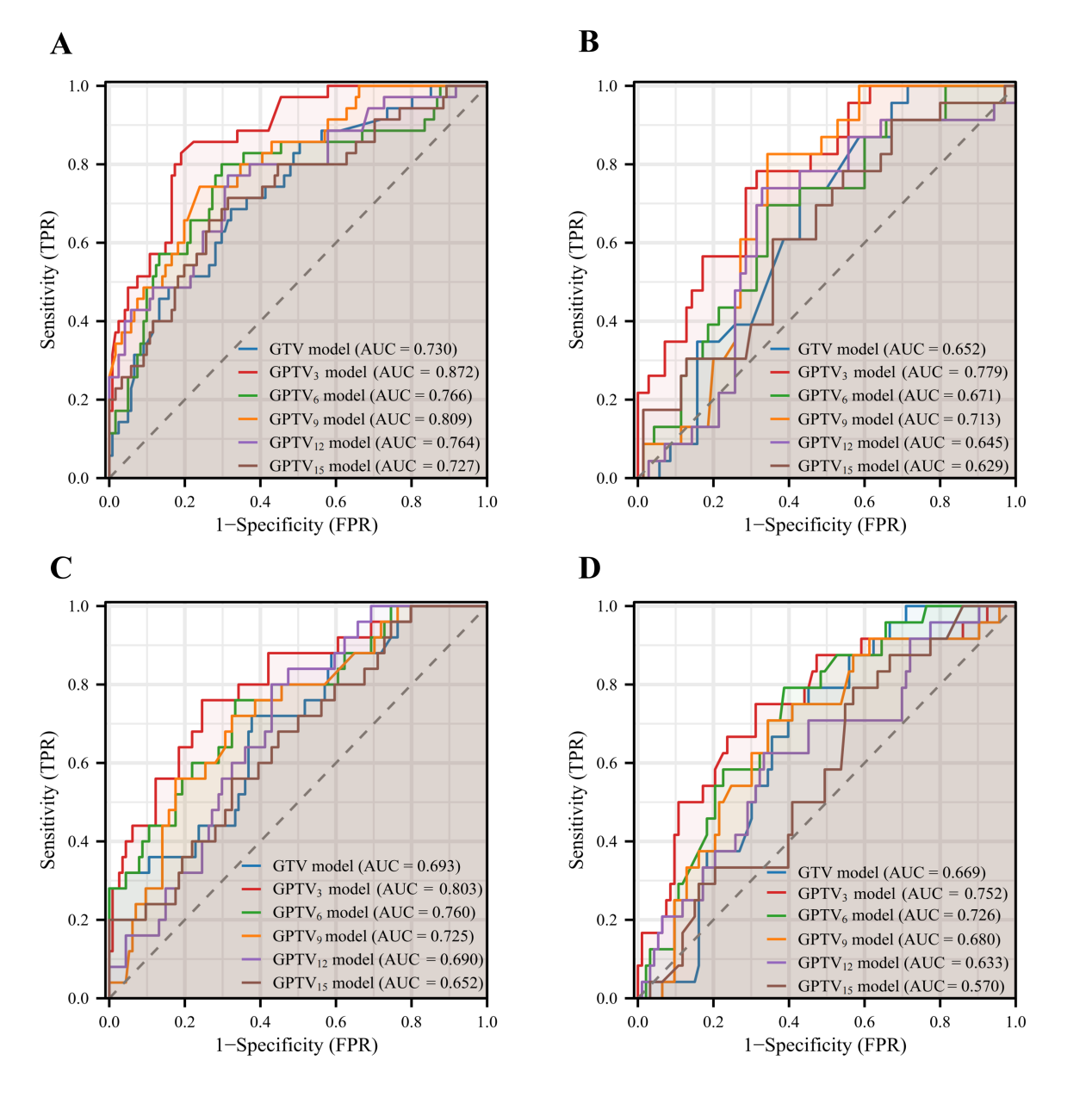
**

**Figure S4.** The comparison of GTV, GPTV_3_, GPTV_6_, GPTV_9_, GPTV_12_, and GPTV_15_ radiomics signatures using Delong tests (*P* value) in the training set (**A**), validation set 1 (**B**), validation set 2 (**C**), and validation set 3 (**D**).

**
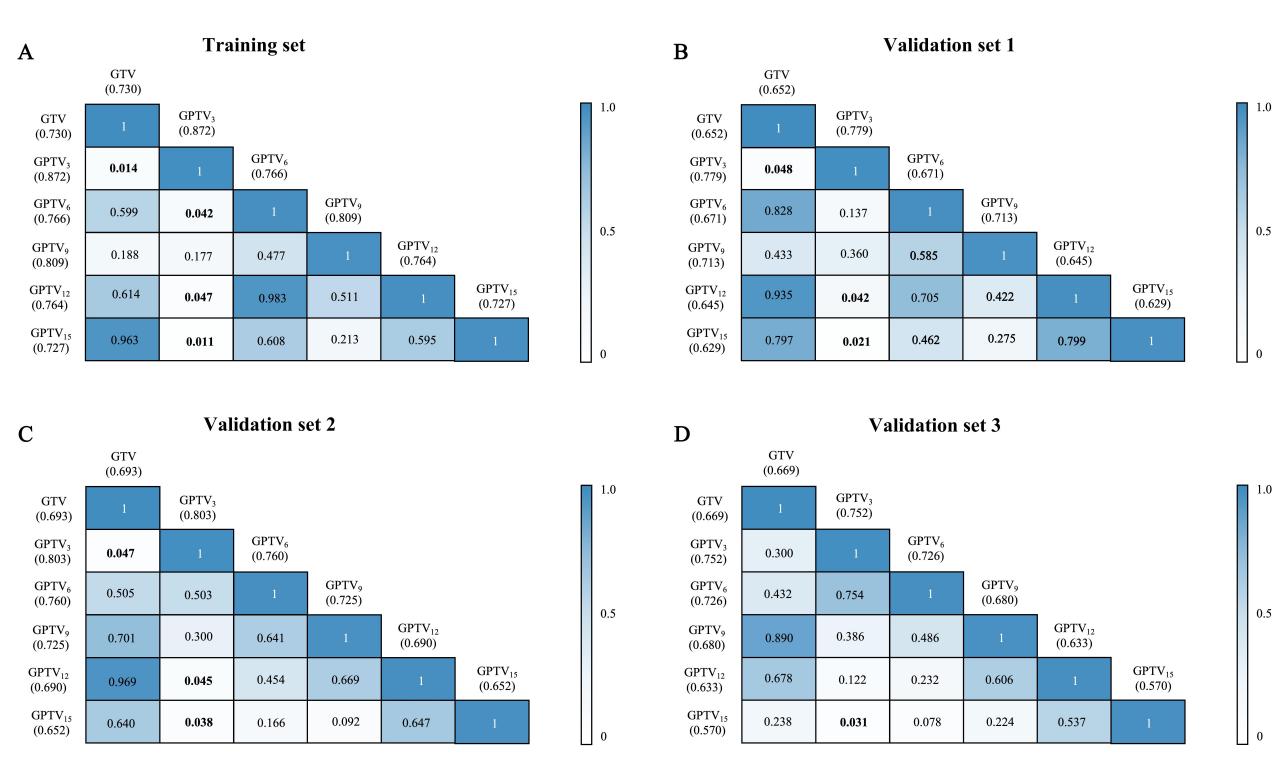
**

**Figure S5.** The performance of GPTV_3_ radiomics signature with six machine learning algorithms in all sets. The ROC curves of six different machine learning models in the training set (**A**), validation set 1 (**B**), validation set 2 (**C**), and validation set 3 (**D**).

**
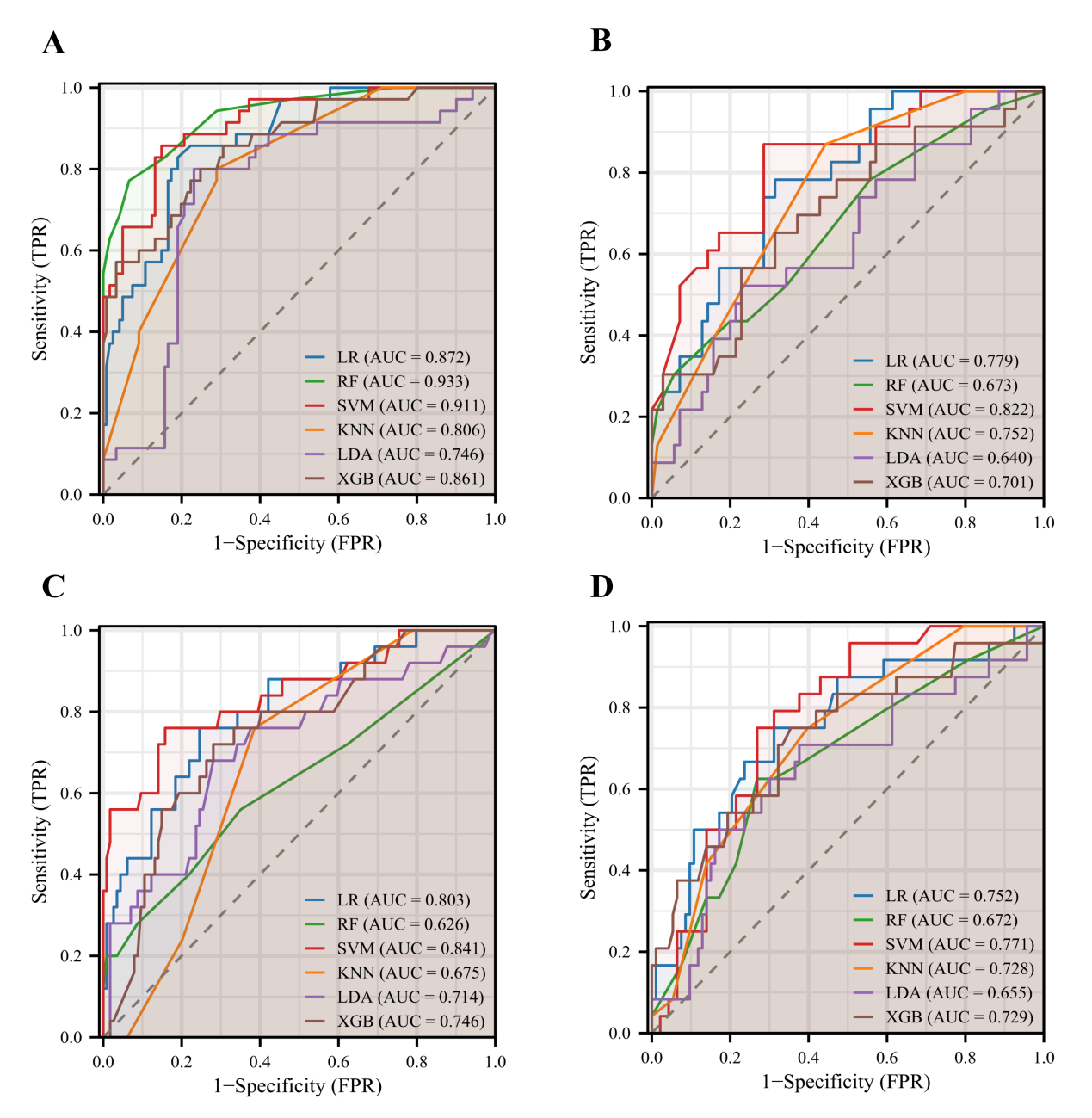
**

**Figure S6.** The comparison of six different machine learning models using Delong tests (*P* value) in the training set (**A**), validation set 1 (**B**), validation set 2 (**C**), and validation set 3 (**D**).

**
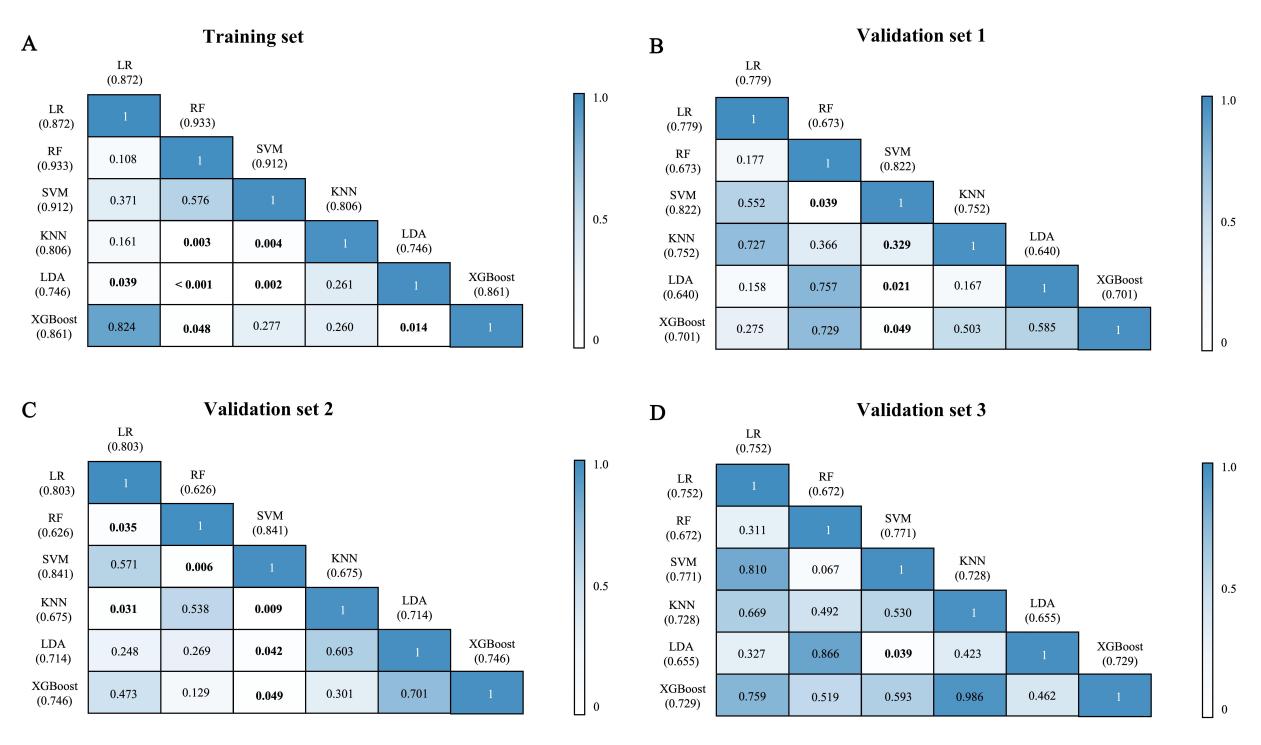
**

**Figure S7.** The nomogram to predict the risk of ALK (+) in patients with lung adenocarcinoma. Draw a line straight upward to the points’ axis to determine the points of each variable, add the points, and locate the final sum on the axis of total points. Finally, draw a line straight down to find the patient’s probability of ALK (+).


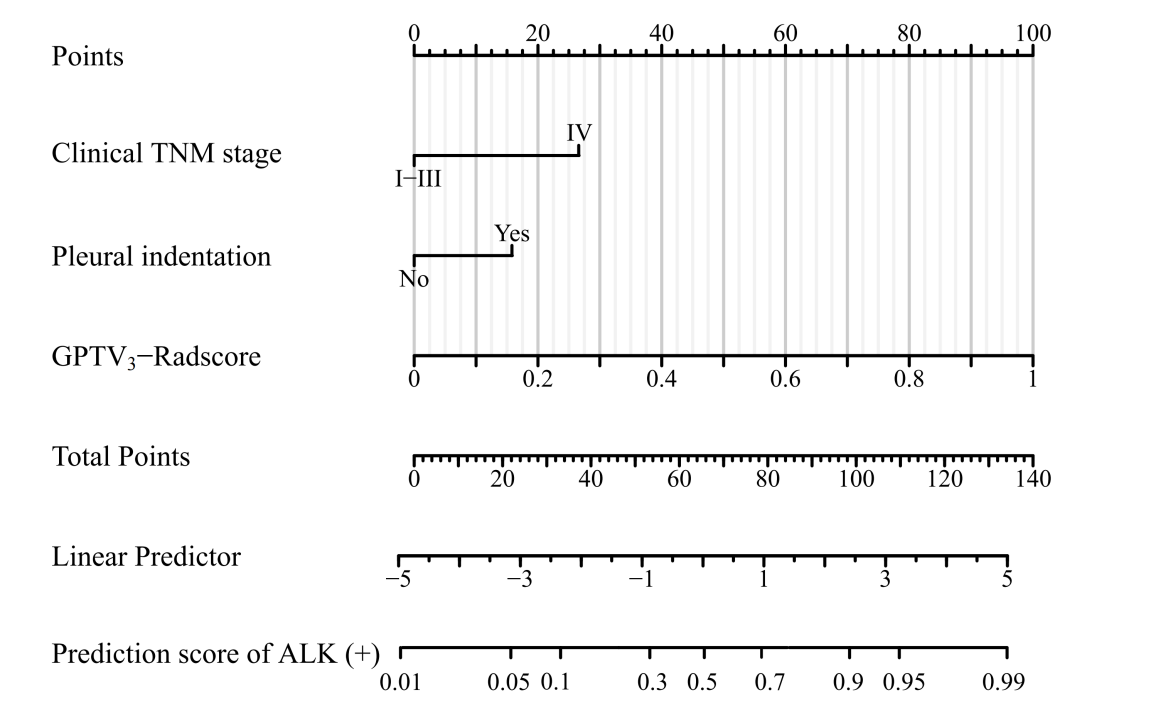


**Figure S8.** The performance of clinical model, GPTV_3_ radiomics model, nomogram was evaluated in the training set. The ROC curves (**A**), calibration curves (**B**), and DCA curves (**C**) of different models.


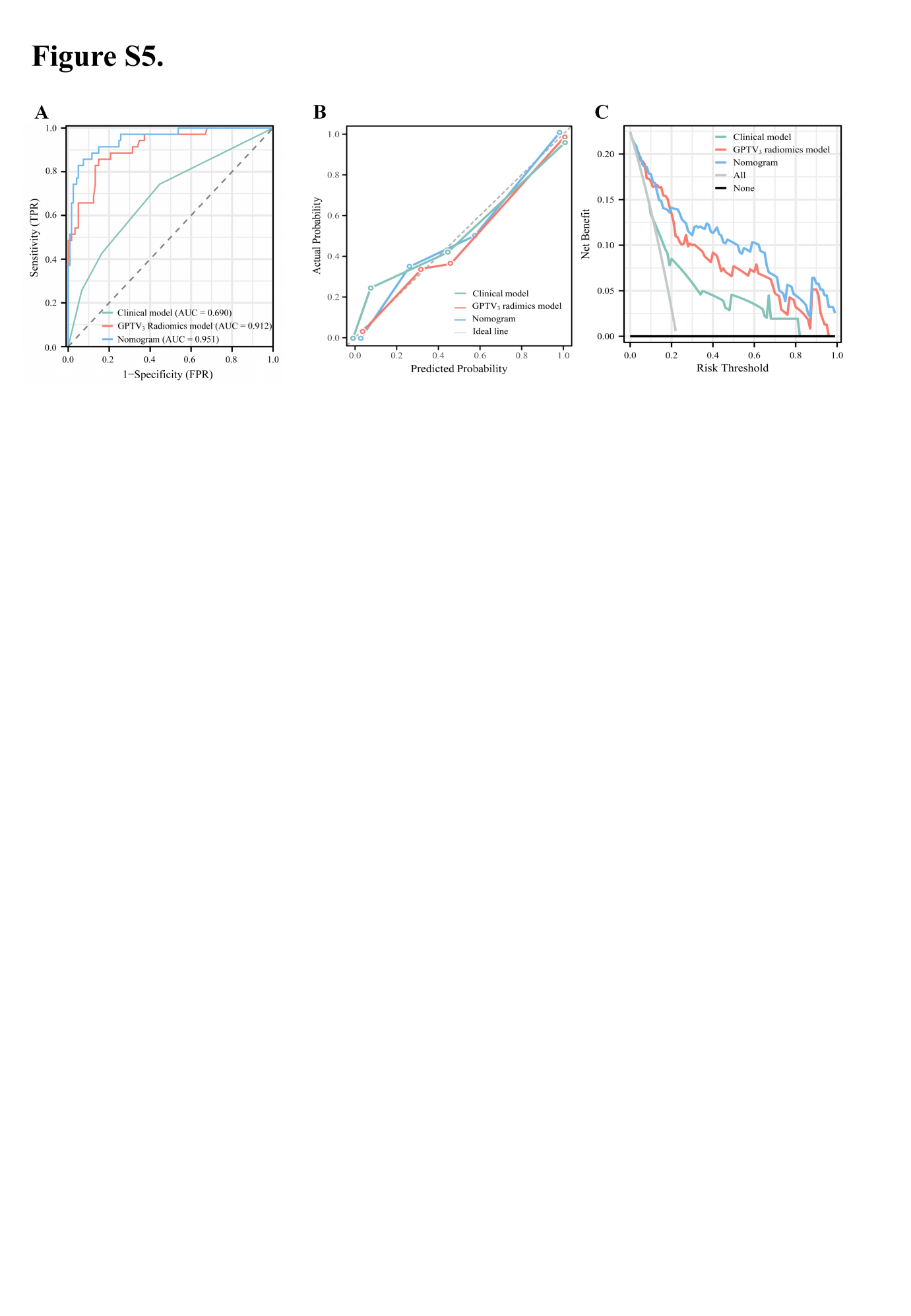


**Figure S9.** AUC of the combined model for predicting ALK (+) in different subgroups stratified by sex (**A, B**) and smoking status (**C, D**). Parentheses indicate 95% CIs for AUCs. AUC, area under the receiver-operating characteristic curve; CI, confidence interval.


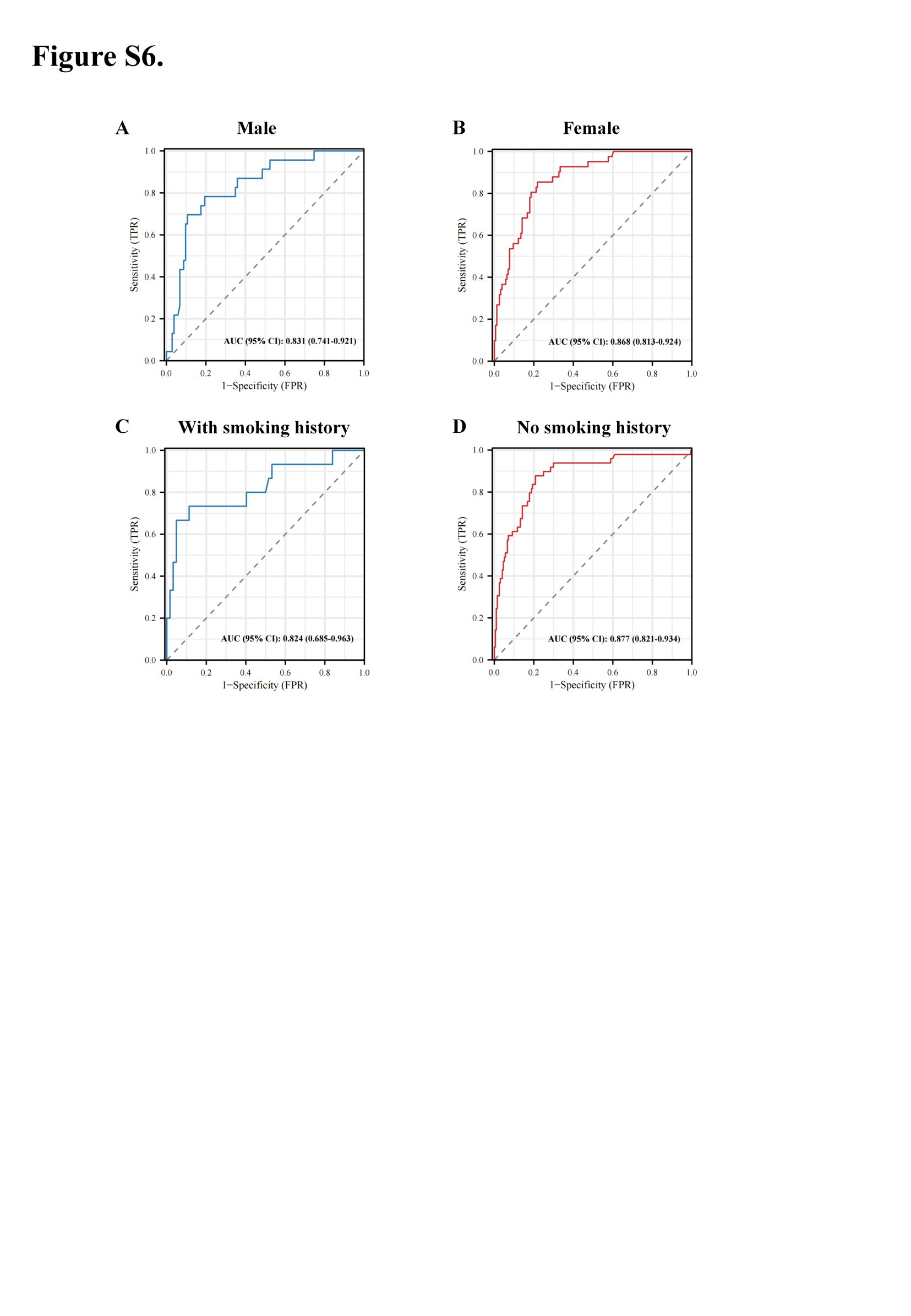

Supplement: Supplementary file 1 — Supplementary Material 1 [file 40644_2025_856_MOESM1_ESM.docx]
